# Reduced IL-10 production by FoxP3^+^IL-10^+^ Treg cells is partially compensated by complosome-associated induction of regulatory FoxP3^−^IL-10^+^ T cells in allergic eosinophilic asthma

**DOI:** 10.1093/cei/uxag017

**Published:** 2026-03-25

**Authors:** Julie Stichova, Peter Slanina, Zita Chovancova, Jan Baros, Marek Litzman, Jiri Litzman, Marcela Vlkova

**Affiliations:** Faculty of Medicine, Institute of Clinical Immunology and Allergology, Masaryk University, Brno, Czechia; Institute of Clinical Immunology and Allergology, St. Anne’s University Hospital, Brno, Czechia; Faculty of Medicine, Institute of Clinical Immunology and Allergology, Masaryk University, Brno, Czechia; Institute of Clinical Immunology and Allergology, St. Anne’s University Hospital, Brno, Czechia; Faculty of Medicine, Institute of Clinical Immunology and Allergology, Masaryk University, Brno, Czechia; Institute of Clinical Immunology and Allergology, St. Anne’s University Hospital, Brno, Czechia; Faculty of Medicine, Institute of Clinical Immunology and Allergology, Masaryk University, Brno, Czechia; Institute of Clinical Immunology and Allergology, St. Anne’s University Hospital, Brno, Czechia; Department of Economics, Faculty of Business and Economics, Mendel University, Brno, Czechia; Faculty of Medicine, Institute of Clinical Immunology and Allergology, Masaryk University, Brno, Czechia; Institute of Clinical Immunology and Allergology, St. Anne’s University Hospital, Brno, Czechia; Faculty of Medicine, Institute of Clinical Immunology and Allergology, Masaryk University, Brno, Czechia; Institute of Clinical Immunology and Allergology, St. Anne’s University Hospital, Brno, Czechia

**Keywords:** asthma, complosome, CD46, CD49b, FoxP3^+^ Treg cells, FoxP3^−^IL-10^+^ T cells

## Abstract

Role of the anti-inflammatory cytokine IL-10 in allergic eosinophilic asthma (AEA) remains controversial, with studies reporting inconsistent findings. Nevertheless, reduced IL-10 production has been described and may contribute to the pathophysiologic features of the disease. Here, we compared FoxP3^+^IL-10^+^ Treg cells with the complosome-CD46-induced regulatory FoxP3^−^IL-10^+^ T cell capacity to produce IL-10 in AEA. We analyzed CD4^+^ T cells from 58 adults with controlled AEA and 49 healthy donors using *ex vivo* phenotypic characterization combined with a defined αCD3/αCD46/IL-2 activation model. CD4^+^ T cells from patients with controlled AEA showed a predominance of memory CD4^+^ T cells, reduced frequencies of FoxP3^+^ Treg cells and increased IFN-γ plasma levels. After CD46-mediated activation *in vitro*, CD4^+^FoxP3^−^ T cells from AEA patients exhibited enhanced IFN-γ production together with altered expression of complosome-associated components, including increased CD46 expression and reduced surface C3bIn parallel, reduced frequencies of CD4^+^FoxP3^+^ Treg cells with impaired IL-10-producing capacity and normal levels of regulatory FoxP3^−^IL10^+^CD49b^+^ T cells were observed in AEA. The inducible regulatory response within the FoxP3^−^ compartment was primarily driven by increased IL-10 secretion at the single-cell level rather than by an increased frequency of IL-10^+^ cells. Together, these findings define *ex vivo* features of complosome-associated CD4^+^ T cell activation in controlled AEA and indicate that impaired FoxP3^+^ T cells with diminished IL-10-producing capacity can be partially compensated by complosome-CD46 driven differentiation of CD4^+^FoxP3^−^ T cells with stronger IL-10 secretion despite overall increase in T1 responses under defined experimental conditions.

## Teaser

The complosome regulates Th1 cell differentiation through autocrine production of C3a and C3b, engaging CD46/C3b signaling, and subsequently promotes the switching into induced regulatory T cells producing IL-10 during the late phase of the adaptive immune response. CD4^+^ T cells from allergic eosinophilic asthma (AEA) patients show elevated CD46 expression together with decreased C3b production that is associated with dysregulated cytokine environment in AEA (↑ IFN-γ, ↑ IL-10). However, upon in vitro αCD3/αCD46/IL-2 activation model, CD4^+^ T-cells from AEA patients differentiate into elevated numbers of induced regulatory T cell subsets, including CD4^+^FoxP3-IL-10^+^LAG-3^+^CD49b^+^ and CD4^+^FoxP3-IL-10^+^LAG-3-CD49b^+^ T cells and these populations secrete large quantities of the anti-inflammatory cytokine IL-10, suggesting a potential candidates modulating chronic inflammation in asthma.

## Graphical Abstract

**Graphical Abstract:**
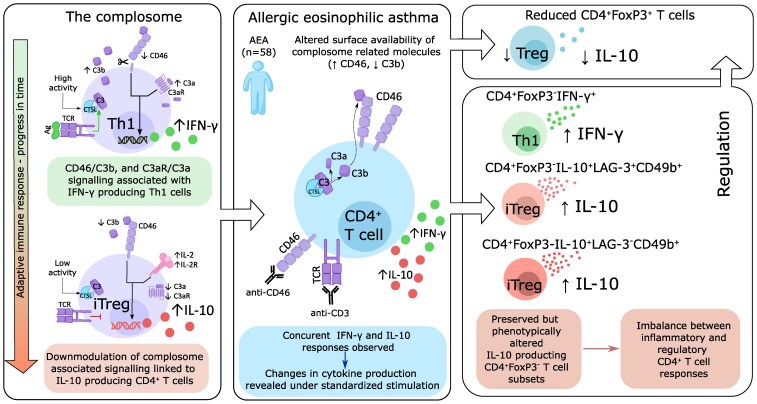

## Introduction

Allergic eosinophilic asthma (AEA) is a heterogeneous disease characterized by chronic airway inflammation and airway hyperresponsiveness resulting in repeated periods of wheezing, shortness of breath, and coughing affecting more than 300 million people globally [1]. The primary pathophysiological mechanism involves chronic T2 response accompanied by dysregulated T cell responses that persist even outside periods of allergen exposure [2, 3]. Although T2-mediated mechanisms dominate acute exacerbations, accumulating evidence shows that patients with stable disease exhibit broader deviations in CD4^+^ T cell homeostasis, including altered memory differentiation, heightened effector responsiveness, and impaired regulatory control [4, 5]. In particular, reduced frequencies or impaired function of forkhead box 3 (FoxP3^+^) regulatory T cells (Tregs) have been repeatedly reported among different asthma phenotypes [6–8] together with documented evidence regarding pathogenic involvement of Th1 cells in both murine models of asthma [9] and in humans [10–13], suggesting that defects in regulatory pathways may contribute to immune activation beyond classical allergen-driven immune response.

Recently, attention has been directed toward CD4^+^ T cell regulation mechanisms, where growing body of evidence highlighted the importance of a liver-independent (intracellular) complement, termed ‘the complosome’ [12, 14–16]. The complosome central molecule is CD46, which controls a metabolic program in human CD4^+^ T cells, comprising differentiation into pro-inflammatory Th1 cells, followed by switching into IL-10-producing induced type 1 regulatory (Tr1) cells [17–20] expressing both lymphocyte activation gene-3 (LAG-3) and CD49b activation markers [21, 22].

The CD46-driven regulatory program [17, 23, 24] predominantly occurs in CD4^+^FoxP3^−^ T cells [25] and is dependent on intracellular C3 stores that are gradually cleaved by cathepsin L to generate autocrine C3b. This C3b subsequently engages CD46 [18], while CD46 internalization dynamics [26] and the availability of IL-2 in the surrounding microenvironment influence the progression of the Th1-to-Tr1 switch. Furthermore, the importance of the complosome in IFN-γ-to-IL-10 cell switching has been underpinned by the finding that this process is impaired in autoimmune disorders with T1 signature, such as multiple sclerosis [15, 19, 27] and rheumatoid arthritis [15, 18]. Additionally, West & Kemper [16] proposed that allergic diseases including asthma could be a promising target for investigating complosome-related pathophysiology, since the role of the complosome in allergic asthma remains largely unexplored.

Several observations suggest that complosome-dependent T cell regulation may be relevant to AEA. First, memory CD4^+^ T cells, frequently expanded in adults with AEA, show enhanced CD46 expression [19, 28] and stronger IL-10 competence than naive CD4^+^ T cells [19], raising the possibility that altered naive/memory T cells proportions may influence CD46-driven regulatory responses. Second, systemic IL-10 levels in asthma show variability among studies [7, 29–31], and the extent to which IL-10 dysregulation reflects impaired regulatory function at the cell level is still unclear. In this context, increased systemic IFN-γ levels reported in a subset of AEA patients [11, 12] may be compatible with a perturbed IFN-γ/IL-10 balance and an altered resolution of Th1 responses, a process that is physiologically linked to CD46-dependent Th1-to-Tr1 switching. Additionally, no study to date has examined *ex vivo* alterations in CD4^+^FoxP3^+^ Tregs and CD4^+^FoxP3^−^ T cells considering the CD46-complosome-IL-10 pathway in AEA.

Therefore, the present study was designed to specifically examine the regulatory phase of CD4^+^ Th1 cell activation in AEA. By combining *ex vivo* phenotypic analysis of CD4^+^ T cell subsets with a mechanistic αCD3/αCD46/IL-2 activation model, we aimed to assess complosome-driven Th1-to-Tr1 switching along with IFN-γ and IL-10 production. To focus on stable immunological features rather than transient allergen-driven responses, analyses were performed on samples obtained from adult AEA patients outside of the pollen season. We further investigated how alterations in CD4^+^ T cell phenotype, CD46/C3b expression, and IFN-γ/IL-10 competence contribute to the balance between IFN-γ−driven effector activity and IL-10–mediated regulation, and whether inducible regulatory pathways may compensate for reduced FoxP3^+^ Treg cells in AEA.

## Methods and materials

### Study participants

We enrolled a total of 58 adult patients with compensated AEA (median age: 40, range: 19−62), primarily allergic to tree and grass pollen, and 49 healthy donors (HDs) with a median age of 35 (range: 21–76). AEA cohort was diagnosed in concordance with the GINA guidelines [32]. All investigated patients had clinically significant allergic symptoms (Supplementary Figure S1). The diagnosis was based on symptoms and an obstructive pattern in the spirometry analysis (airway hyperresponsiveness was defined by a ≥12% increase in forced expiratory volume in 1 second (FEV1) or a ≤20% fall in FEV1 during provocation with methacholine, accompanied by an increased concentration of exhaled nitric oxide >35 ppb). Additionally, elevated plasma levels of eosinophilic cationic protein (ECP), eosinophils, and total/specific IgE for aleroallergens were present (Supplementary Figure S1). All patients were treated with inhaled corticosteroids primarily in combination with long-acting beta agonists at the time of blood sample collection (Supplementary Table S2), which was performed between September and February. We excluded patients who had received oral corticosteroid treatment at least 6 months prior to study initiation or who had previously undergone an allergen immunotherapy. AEA patients who had received biological therapy were excluded from the study as well. The cohort of HDs exhibited no clinical or laboratory symptoms of allergy or atopy and were free of any known diseases (Supplementary Table S2). Those HDs who later showed laboratory signs of atopy (increased total and specific IgE) were excluded from the study, with a final count of 49 HDs. All AEA patients and HDs signed an informed consent form to participate in this study, and their anonymity was preserved using methods approved by the Institutional Ethics Committee, approval number 34/2018. The study has been performed in accordance with the Declaration of Helsinki.

### Evaluation of total IgE, specific IgE, ECP, eosinophils, and CD4^+^ T cell subsets

Total IgE serum levels were determined using the Immage-800 (Beckman Coulter, Brea, CA, USA). Specific IgE and ECP were measured with the Immulite 2000 Xpi (Siemens Healthineers AG, Forchheim, Germany). The percentage and absolute count of eosinophils were quantified using Sysmex XN-3000 (Sysmex Corporation, Kobe, Japan) from peripheral blood. CD4^+^ T cell subsets from peripheral blood were stained with Dry Reagent Tubes DURAClone IM T Cell Subsets Tube (Beckman Coulter, Bengalúru, India) and custom-designed DEFT2-Treg Tubes via Dry reagents service from Exbio (Prague, Czechia, Supplementary Table S3) and analyzed by DX Flex flow cytometer (Beckman Coulter, Brea, CA, USA). Basic gating strategy is outlined in Supplementary Figure S2a. Surface CD46 expression on naive and memory CD4^+^ T cell subsets was measured using staining protocol A (Supplementary Table S3) and analyzed by Navios EX flow cytometer (Beckman Coulter, Brea, CA, USA).

### Cell isolation

Peripheral blood mononuclear cells (PBMCs) were purified from 20 ml of venous blood collected into K_3_EDTA tubes (Sarstedt S-monovette) using standard Ficoll-Paque gradient centrifugation according to the manufacturer’s instructions (Amersham Pharmacia, Upsala, Sweden). The plasma from the upper gradient layer was collected and frozen at −80°C, while the PBMCs were cryopreserved in freezing media containing fetal bovine serum (FBS; Sigma-Aldrich Chemie GmbH, Steinheim, Germany) and 10% dimethyl sulfoxide (DMSO, Sigma-Aldrich Chemie GmbH, Steinheim, Germany) and stored at −80°C. Later, the cryopreserved samples were thawed as previously described [33]. In order to prevent PBMCs clumping, samples were treated with DNase I (10 μg/ml; Roche Diagnostics GmbH, Mannheim, Germany). Next, PBMCs were washed in phosphate-buffered saline (PBS) and resuspended in isolation medium (PBS containing 2% FCS and 1 mM EDTA). Subsequently, CD4^+^ T cells were isolated using the EasySep^TM^ Human CD4^+^ T cell isolation kit (STEMCELL Technologies, Inc., Vancouver, Canada) by following the standard manufacturer procedure. The isolated CD4^+^ T cells were resuspended in complete RPMI 1640 medium (Sigma-Aldrich, Saint Louis, MO, USA) supplemented with L-glutamine (2 mM), penicillin (50 U/ml), streptomycin (50 μg/ml) (Sigma-Aldrich Chemie GmbH), and 10% FBS and counted. The purity and viability of the CD4^+^ T cells (typically >91% and >95%, respectively) were assessed by flow cytometry.

### CD4^+^ T cell culture for flow cytometry

Isolated CD4^+^ T cells were cultured in complete RPMI 1640 medium at a density of 7.5 × 10^5^ cells/ml in 96-well round-bottomed culture plates (Thermo Fisher Scientific, Waltham, MA, USA) and stimulated using αCD3/αCD46/IL-2 or αCD3/αCD28/IL-2 (Table 1). CD4^+^ T cells were maintained in the absence or presence of the stimuli in 5% CO_2_ and 37°C for 60 hours. After that time, Brefeldin A (REF B7651, Sigma-Aldrich Chemie GmbH) was added into the wells in final concentration of 1 μg/ml for an additional 4 hours.

**Table 1:** Stimuli used for CD4^+^ T cell activation *in vitro*.

Stimulus	Work concentration	Form	Clone	Manufacturer	REF	Literature research
αCD3	10 μg/ml	Coated in wells	HIT3a	Biolegend	3000332	Astier et al. [51] Kemper et al. [20]
αCD46	5 μg/ml	Coated in wells	MEM-258	Exbio	11-342-C100	Kemper et al. [20]. Xu et al. [7]
αCD28	5 μg/ml	Coated in wells	CD28.2	Invitrogen	16-0289-85	Kemper et al. [20] Xu et al. [7]
IL-2	50 U/ml	Soluble	*E. coli*	PeproTech	200-02	Cardone et al. [15]

### Flow cytometry

Nonactivated (NA) and activated CD4^+^ T cells were stained for flow cytometry according to the Supplementary Table S3. Samples for surface staining and cytokine production (Protocol B) were stained according to the Affymetrix eBioscience intracellular antigens staining procedure A for 96-well plate (eBioscience^TM^ Intracellular Fixation & Permeabilization Buffer Set). Samples for Ki-67 (Protocol C) and FoxP3 (Protocol D) detection were stained according to the Affymetrix eBioscience intracellular antigens staining procedure B for 96-well plates (eBioscience^TM^ FoxP3/Transription Factor Staining Buffer Set). CD4^+^ T cell activation status was measured using Navios EX cytometer (Beckman Coulter, Brea, CA, USA) with a basic gating strategy depicted in Supplementary Figure S2b. Data were analyzed using Kaluza software (Beckman Coulter, Brea, CA, USA).

### ELISpot

For IFN-γ and IL-10 evaluation by ELISpot assay, we used ELISpot Flex Human IFN-γ kit, ELISpot Flex Human IL-10 kit and MultiScreen® MSIPS4510 Filter plates from Millipore® all from MABTECH AB, Sweden. CD4^+^ T cells (7.5 × 10^5^ cells/ml) were preincubated in 96-well round-bottomed plates without stimulation or with αCD3/αCD46/IL-2 (Table 1) and cultivated for 24 hours. The following day, preactivated CD4^+^ T cells were washed with PBS and resuspended in the complete RPMI 1640 medium with a new dose of soluble αCD3, αCD46 and IL-2, and immediately transferred into precoated ELISpot wells. CD4^+^ T cells were cultivated in 5% CO_2_ and 37°C for an additional 40 hours to match the end of the incubation period with cells seeded for flow cytometry. After that, cells were discarded, and ELISpot plates were developed according to the manufacturer’s procedure. For development, we used BCIP/NBT-plus substrate (REF 3650-10, MABTECH AB, Sweden), which was filtered through 0,45μm filter (FiltraTECH, Fisher Scientific, Czech Republic). For standard development of all plates, the incubation time with the substrate was estimated to be exactly 6 minutes. For plate evaluation, we used ImmunoSPOT reader with ImmunoSPOT software version 5.4.0.4 (Cellular Technology Limited, Cleveland Ohio, USA). IFN-γ and IL-10 responses were averaged across duplicates, and results are presented as spot forming unit)/million CD4^+^ T cells and total area covered by spots as 10^−3^ square millimeter. Representative pictures of ELISPOT IFN-γ and IL-10 production from one HD are shown in Supplementary Figure S3a.

### ELISA

ELISAs were performed from cell culture supernatants (SNs) collected after 64 hours of CD4^+^ T cell stimulation. Concentrations of IFN-γ and IL-10 were measured with the Human IL-10 ELISA MAX^TM^ Deluxe set and IFN-γ ELISA MAX Deluxe set (both from Biolegend, San Diego, CA, USA) with analytical sensitivities of 2 and 4 pg/ml, respectively. Concentrations of IFN-γ and IL-10 in plasma samples were measured with the IFN-gamma Human ELISA kit, High Sensitivity (Thermo Fisher Scientific, Waltham, MA, USA) and Human IL-10 ELISA kit (RayBiotech Life Inc., Peachtree Corners, GA, USA) with analytical sensitivities of 0.06 and 1.00 pg/ml, respectively. Concentration of soluble CD46 (sCD46) in plasma was estimated using human membrane cofactor protein/CD46 ELISA kit (Elabscience Biotechnology Inc. Houston, TX, USA) with analytical sensitivity of 4.69 pg/ml. C3b and soluble IL-2R α chain (sIL-2RA) in cell culture SNs were assessed using Human Complement C3b ELISA kit (Abcam, Cambridge, UK) and human IL-2RA/CD25 ELISA Kit (Thermo Fisher Scientific, Waltham, MA, USA) with analytical sensitivities of 57 and 15.00 pg/ml, respectively.

### Statistical analysis

Statistical analysis was performed using GraphPad Prism version 5.0 (GraphPad Software, San Diego, CA, USA). Data were tested by Shapiro–Wilk test for normal distribution with a significance level α set to default 0.05. Data that passed the normality test were analyzed by unpaired *t*-test or one-way ANOVA test with a Turkey correction where appropriate. Data that did not pass the normality test were analyzed using the nonparametric Mann–Whitney *U* test or Kruskall–Wallis test with Dunn’s correction where appropriate *P*-values <0.05 were considered as a measure of statistical significance.

## Results

### AEA patients exhibit an increased frequency of memory CD4^+^ T cells and reduced CD4^+^ Tregs in peripheral blood

**Figure 1:**
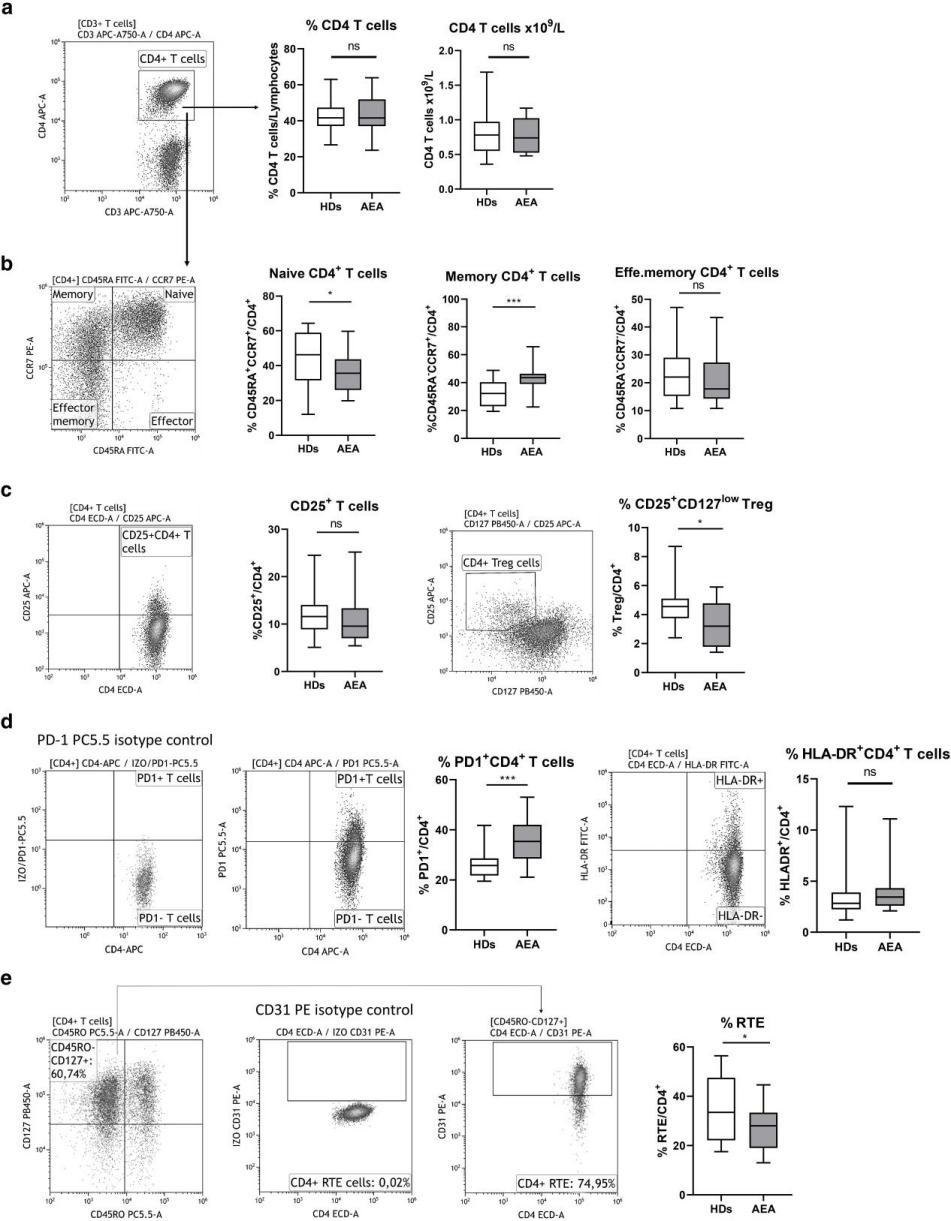
Peripheral blood CD4^+^ T cell subsets. (a) CD4^+^ T cells from peripheral blood were gated from CD3^+^ T lymphocytes. The relative percentage and absolute count (×10^9^/L) of CD4^+^ T cells was similar in both HDs and AEA patients. (b) Next, CD4^+^ T cells were further divided into naive, memory, and effector subsets based on CD45RA and CCR7 expression. AEA patients exhibited a reduced proportion of naive CD4^+^ T cells, with a concomitant increase in memory CD4^+^ T cells. (c) Treg cells were identified by their expression of CD25 and low levels of CD127. AEA patients exhibited a lower percentage of Treg cells compared to HDs. (d) HLA-DR expression on CD4^+^ T cells remained within physiological levels in AEA patients, whereas PD1 expression was increased. (e) Recent thymic emigrants (RTE) were evaluated as CD4^+^CD45RO^−^CD127^+^CD31^+^ cells. Dot plots are representative of one healthy donor, and data are presented as box & whiskers (median and min-max). Statistical analysis was performed using an unpaired T-test. ns (not significant), *P ≤ 0.05, ***P ≤ 0.001. HDs, healthy donors; AEA, allergic eosinophilic asthma.

First, differences in the distribution of CD4^+^ T cell subsets in peripheral blood between HDs and AEA patients were investigated (Fig. 1). Although the absolute and relative counts of CD4^+^ T cells were within the normal range (Fig. 1a), AEA patients demonstrated a significant reduction in naive T cells (*P* < 0.033), accompanied by an increase in the memory T cell compartment (*P* = 0.0009, Fig. 1b). Additionally, AEA patients showed decreased frequency of Treg cells (*P* = 0.029, Fig. 1c), significantly increased PD-1 expression (*P* = 0.0005, Fig. 1d). The percentage of HLA-DR expression was within a normal range in AEA patients (Fig. 1d), whereas the proportion of recent thymic emigrants (RTE, *P* = 0.041) was decreased in AEA patients (Fig. 1e).

### CD4^+^ T cells from AEA patients show increased CD46 expression

**Figure 2:**
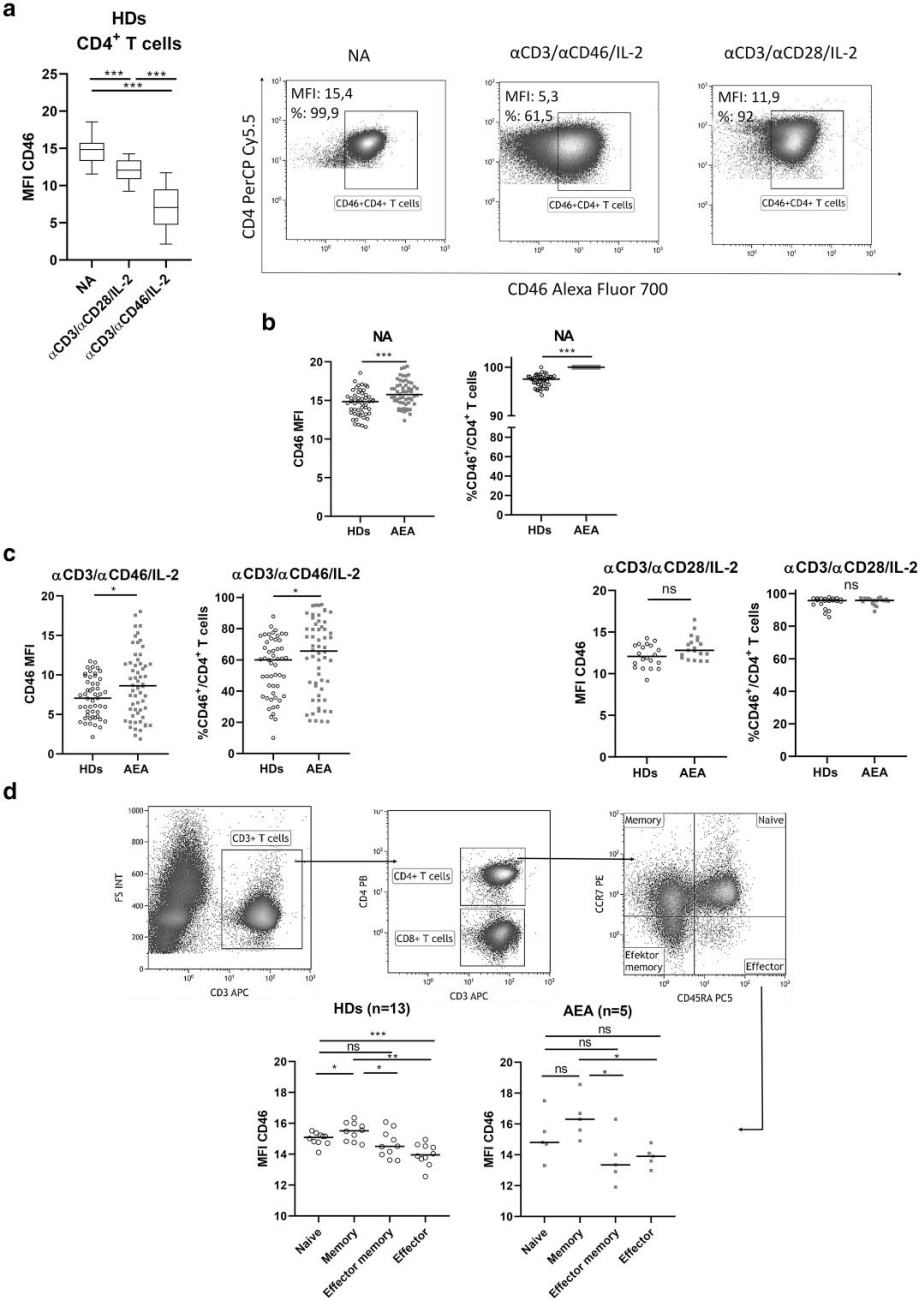
Surface CD46 expression on CD4^+^ T cells and CD4^+^ T cell subsets. (a) Surface CD46 expression on CD4^+^ T cells was evaluated in HDs group. The highest expression was detected on NA CD4^+^ T cells, which exhibited a mild reduction following stimulation with αCD3/αCD28/IL-2 and a more pronounced decrease after αCD3/αCD46/IL-2. Data are presented as box & whiskers (median and min-max). Statistical analysis was conducted using an ordinary one-way ANOVA. (b) In AEA patients, an increased CD46 expression was observed on NA CD4^+^ T cells, accompanied by an insufficient downregulation following αCD3/αCD46/IL-2 stimulation. This result was evident in both the CD46 median intensity fluorescence (MFI) and the proportion of CD4^+^CD46^+^ T cells. (c) However, the downregulation of CD46 after αCD3/αCD28/IL-2 stimulation was comparable between HDs and AEA patients. Data were evaluated using an unpaired T-test. (d) Surface CD46 expression was assessed on CD4^+^ T cell subsets from the peripheral blood of a small group of HDs (n = 13) and AEA patients (n = 5). CD4^+^ T cells were categorized into naive, memory, effector memory, and effector T cells based on CD45RA and CCR7 expression. Among these subsets, memory CD4^+^ T cells exhibited the highest CD46 expression followed by naive > effector memory > effector CD4^+^ T cells on both studied groups. Dot plots are representative of one healthy donor. Statistical analysis was conducted using an ordinary one-way ANOVA. The horizontal bar in graphs represents the median. ns (not significant), *P ≤ 0.05, ***P ≤ 0.001. HDs, healthy donors; NA, nonactivated; AEA, allergic eosinophilic asthma.

**Figure 3:**
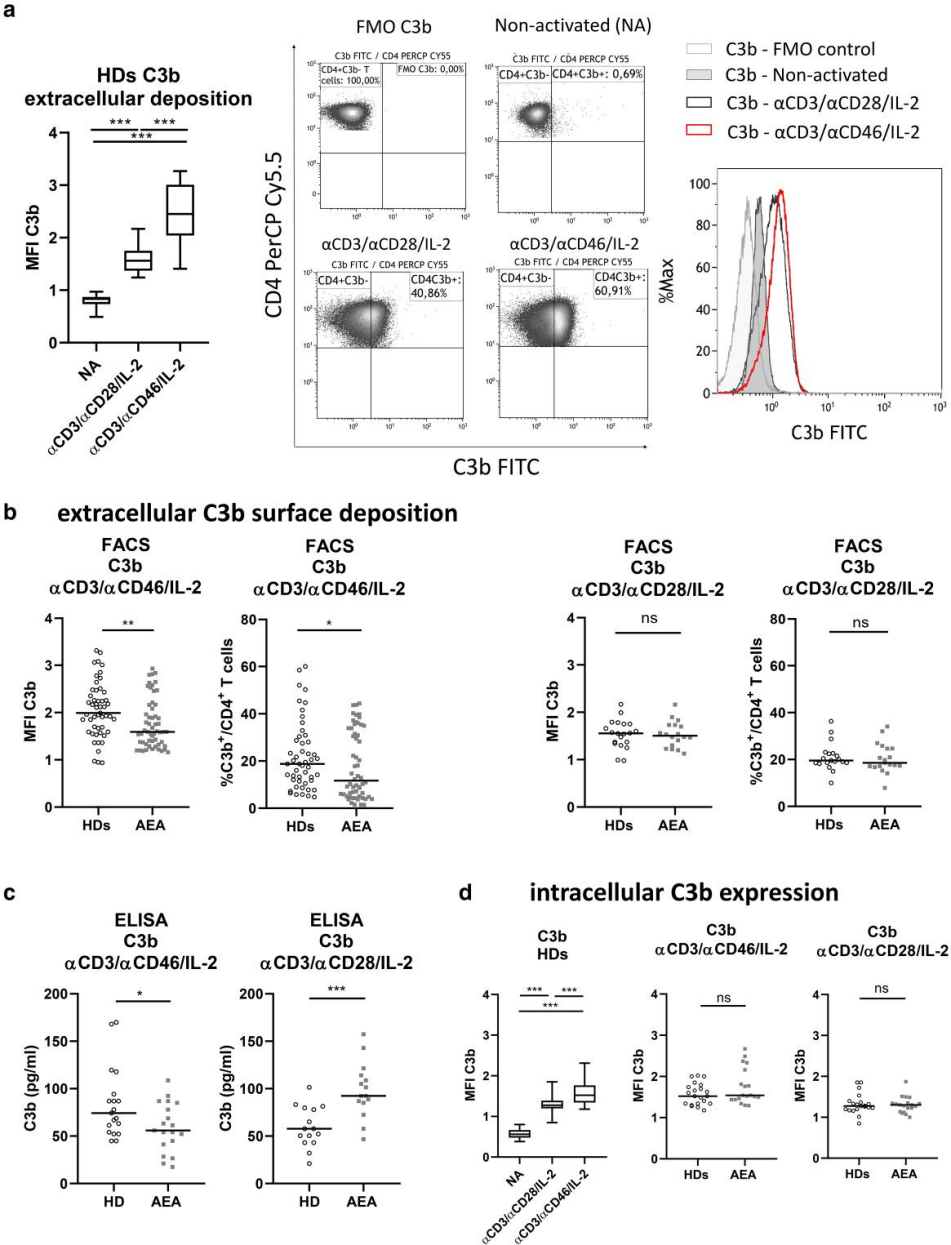
C3b deposition at CD4^+^ T cell surface after activation *in vitro*. (a) Activated CD4^+^ T cells from HDs produced the complement fragment C3b, with a greater production observed under αCD3/αCD46/IL-2 stimulation compared to αCD3/αCD28/IL-2 stimulation. Data are presented as box & whiskers (median, min-max). Representative dot-plots and histograms depict fluoresncence minus-one (FMO) control for C3b and C3b extracellular expression on nonactivated and activated CD4^+^ T cells. (b) The deposition of the C3b fragment on the cell surface was reduced in AEA patients following αCD3/αCD46/IL-2 stimulation. Contrary to that, C3b fragment was deposited similarly to that in HDs following αCD3/αCD28/IL-2 stimulation. These results were observed in both median intensity fluorescence (MFI) and proportion of CD4^+^C3b^+^ T cells. (c) C3b concentration in αCD3/αCD46/IL-2 cell culture SNs was also lower in AEA patients, but increased after αCD3/αCD28/IL-2 stimulation when compared with HDs. (d) We also measured intracellular C3b levels and found that intracellular stores were comparable between HDs and AEA patients following both types of stimulation. The horizontal bar in graphs (b–d) represents the median and graphs with 3 data sets were analyzed using a nonparametric Kruskall–Wallis test, while those with 2 data sets were analyzed using a nonparametric Mann–Whitney *U* test. The αCD3/αCD28/IL-2 stimulation condition, along with C3b quantification via ELISA and intracellular C3b detection, was assessed in a cohort of 19 HDs and 20 AEA patients with moderate AEA. ns (not significant), *P ≤ 0.05, **P ≤ 0.01, ***P ≤ 0.001. HDs, healthy donors; AEA, allergic eosinophilic asthma; SNs, supernatants.

CD46 represents a central complosome-related molecule, acting as a regulator of CD4^+^ T cell metabolism and differentiation into Th1 cells. Therefore, we next examined the expression of surface CD46 on NA and *in vitro* stimulated CD4^+^ T cells (Fig. 2). As expected, CD46 expression decreased after stimulation with αCD3/αCD28/IL-2 (*P* < 0.0001) and even more after αCD3/αCD46/IL-2 (*P* < 0.0001) in HDs (Fig. 2a). However, CD46 expression was increased on NA CD4^+^ T cells from AEA patients in both MFI (*P* = 0.0002) and proportion of CD46^+^ T cells from CD4^+^ T cells (*P* < 0.0001) (Fig. 2b). Less pronounced downregulation of CD46 was observed in AEA patients following αCD3/αCD46/IL-2 stimulation (MFI *P* = 0.01, percentage of CD46^+^/CD4^+^ T cells *P* = 0048) in comparison with HDs (Fig. 2c). Interestingly, CD4^+^ T cells from AEA patients showed normal expression of CD46 within αCD3/αCD28/IL-2 stimulation (Fig. 2c). Intracellular CD46 expression was also comparable between HDs and AEA, as well as soluble CD46 concentration in plasma (Supplementary Figure S4). Given the increased frequency of memory CD4^+^ T cells observed in AEA patients, we further verified whether surface CD46 expression differed between naive and memory CD4^+^ T cells based on CD45RA and CCR7 expression (Fig. 3d). We observed that CD45RA^−^CCR7^+^ memory T cells exhibited the highest CD46 expression in both HDs and AEA patients (*P* = 0.049), followed by CD45RA^+^CCR7^+^ naive >CD45RA^−^CCR7^−^ effector memory >CD45RA^+^CCR7^−^ effector subsets.

### Autocrine C3b is produced less by activated CD4^+^ T cells following αCD3/αCD46/IL-2 stimulation in AEA patients

In a resting state, CD4^+^ T cell metabolism and survival are maintained by tonic autocrine C3b production, which engages the extracellular domain of CD46. This mechanism is also preserved during Th1 cell differentiation, albeit at a markedly elevated rate. Given that the CD46 was increased on CD4^+^ T cells from AEA patients, we further investigated whether the production and surface deposition of its ligand C3b occur normally (Fig. 3). As expected, we observed that C3b was deposited at the cell surface significantly after stimulation in HDs (*P* > 0.0001, Fig. 3a). However, AEA patients exhibited a lower C3b surface deposition (Fig. 3b) when analyzed as C3b MFI (*P* = 0.0028) and as proportion of C3b^+^ T cells from CD4^+^ T cells (*P* = 0.049), as well as decreased C3b production into cell culture SNs (Fig. 3c, *P* = 0.046) specifically after αCD3/αCD46/IL-2 stimulation. Interestingly, following αCD3/αCD28/IL-2 stimulation, C3b was deposited at the cell surface normally (both C3b MFI and percentage of CD4^+^C3b^+^ T cells, Fig. 3b) and production into cell culture SNs was even increased in AEA patients (Fig. 3c, *P* = 0.0002). These results indicate a possible dysregulation in C3b production that might be limited specifically to the CD46 signaling pathway. Therefore, we also assessed intracellular resources of C3b. After activation, intracellular C3b was increased in HDs group (Fig. 3d, *P* < 0.0001) as expected. However, intracellular production of C3b in CD4^+^ T cells was comparable between HDs and AEA patients under both αCD3/αCD46/IL-2 and αCD3/αCD28/IL-2 co-stimulatory conditions (Fig. 3d), indicating no alterations in C3b biosynthesis.

### Activated CD4^+^ T cells from AEA patients produce higher amounts of IFN-γ, IL-10, and soluble IL-2RA

**Figure 4:**
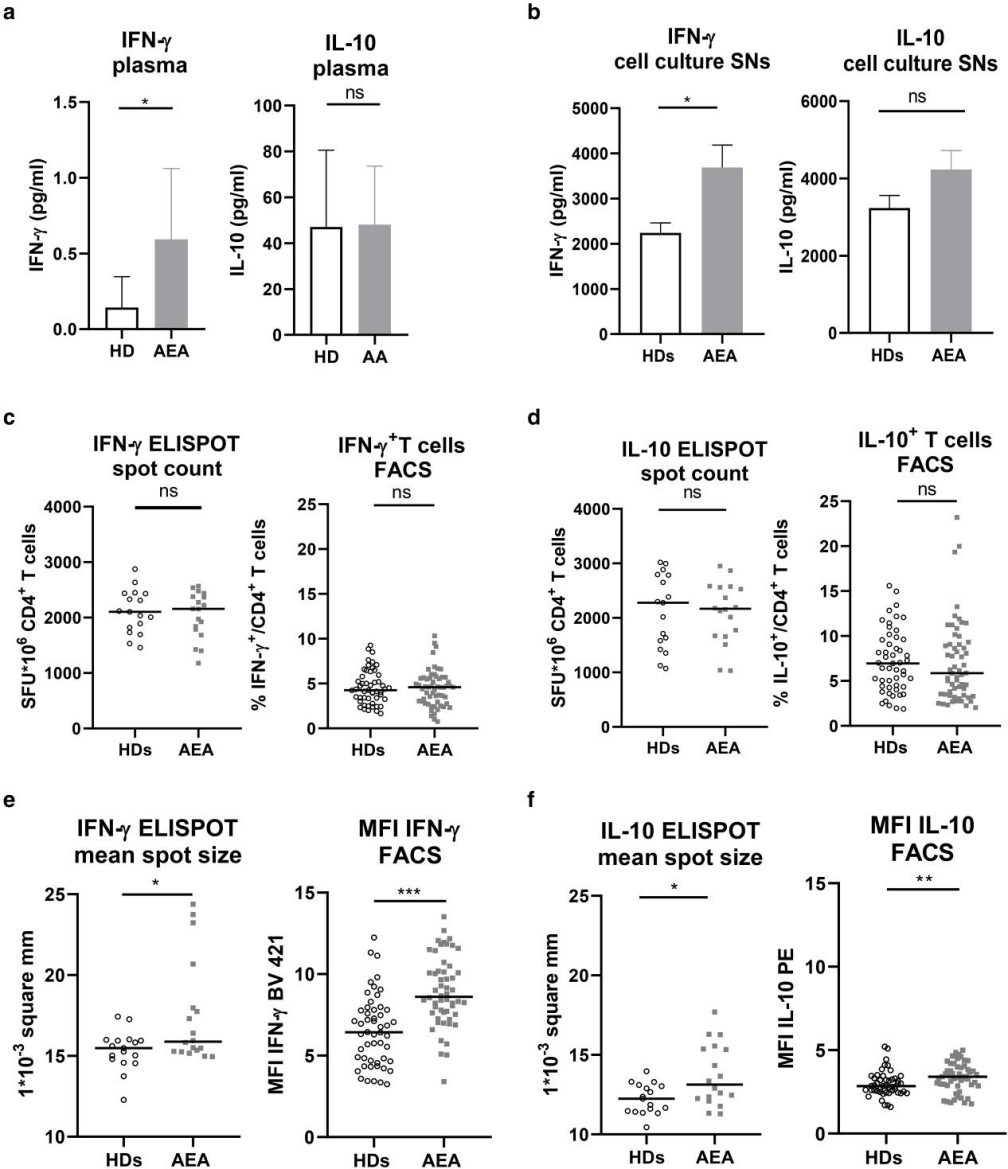
IFN-γ and IL-10 production by CD4^+^ T cells activated through αCD3/αCD46/IL-2. (a, b) IFN-γ concentration measured in plasma and cell culture SNs was increased in AEA patients, while IL-10 concentration in both plasma and cell culture SNs was comparable between HDs and AEA patients. Data are presented as a mean with SEM. (c) The percentages of CD4 ^+^ IFN-γ^+^ T cells were comparable between HDs and AEA patients as well as spot count analyzed by ELISpot. (d) Identical results were observed within CD4^+^IL-10^+^ T cells. (e,f) However, AEA patients exhibited an increased MFI of IFN-γ BV421 and IL-10 PE within both analyzed subsets as well as IFN-γ and IL-10 spot count, indicating enhanced cytokine production in AEA patients. The horizontal bars in graphs c-f represent the median. Data were analyzed using a nonparametrical Mann–Whitney U test. ns (not significant), *P ≤ 0.05, **P ≤ 0.01, ***P ≤ 0.001. HDs, healthy donors; AEA, allergic eosinophilic asthma; SEM, standard error of the mean; SNs, supernatants; MFI, median fluorescence intensity; BV421, brilliant violet 421; PE, phycoerithrine; ELISPOT, enzyme-linked immuno-SPOT assay; SFU, spot-forming unit.

Considering present abnormalities in central complosome-related molecules, CD46 and autocrine C3b in AEA patients following αCD3/αCD46/IL-2 stimulation, we next investigated the capacity of CD4^+^ T cells to produce the signature Th1 and Tr1 cytokines, IFN-γ and IL-10, respectively (Fig. 4). We noticed that IFN-γ concentration was increased (*P* = 0.049), while IL-10 levels in plasma were comparable between HDs and AEA patients (Fig. 4a). IFN-γ levels were also elevated in cell culture SNs (*P* = 0.046), but there was no difference in IL-10 concentration between HDs and AEA patients (Fig. 4b). When analyzed by ELISpot and flow cytometry, no differences were observed in spot count for IFN-γ and IL-10 as well as proportion of IFN-γ^+^ and IL-10^+^ CD4^+^ T cells (Fig. 4c and d). Flow cytometry analysis of cytokine expression within FoxP3^+^ and FoxP3^−^ T cells similarly revealed no difference in the proportion of IFN-γ^+^ T cells between HDs and AEA patients. In contrast, the proportion of CD4^+^FoxP3^+^IL-10^+^ T cells was reduced in AEA patients, indicating not only a decreased FoxP3^+^ T cell compartment, but also their altered function, manifested by decreased IL-10 secretion (Supplementary Figure S3b). Despite comparable spot numbers, ELISpot analysis demonstrated that overall spot size for both IFN-γ and IL-10 was larger in AEA patients (*P* < 0.048, Fig. 4c and d), suggesting enhanced cytokine production per cell, consistent with cytokine concentration measured in cell culture SNs. Flow cytometry analysis supported these findings, since AEA patients exhibited a significantly increased median intensity fluorescence for IFN-γ (Fig. 4e, *P* > 0.002) and IL-10 (*P* = 0.006, Fig. 4f) when compared with HDs, further supporting the stronger cytokine production per cell in AEA patients. Representative flow cytometry data demonstrating increased MFI for IFN-γ and IL-10 in AEA patients are shown in Supplementary Figure S3a.

We also observed a trend towards increased soluble IL-2RA (sCD25) levels in plasma (*P* = 0.06) and increased SNs in cell culture in AEA patients (*P* = 0.0002, Supplementary Figure S5a) together with increased surface CD25 expression on CD4^+^ T cells (*P* = 0.048). However, the proliferation was decreased in CD4^+^ T cells (*P* = 0.0018) (Supplementary Figure S5b) and among IFN-γ^−^IL-10^−^, IFN-γ^+^IL-10^−^, IFN-γ^+^IL-10^+^, and IFN-γ^−^IL-10^+^ T cell subsets in AEA (*P* < 0.0026, Supplementary Figure S5c).

### Activated CD4^+^ T cells from AEA patients show decreased LAG-3 expression

**Figure 5:**
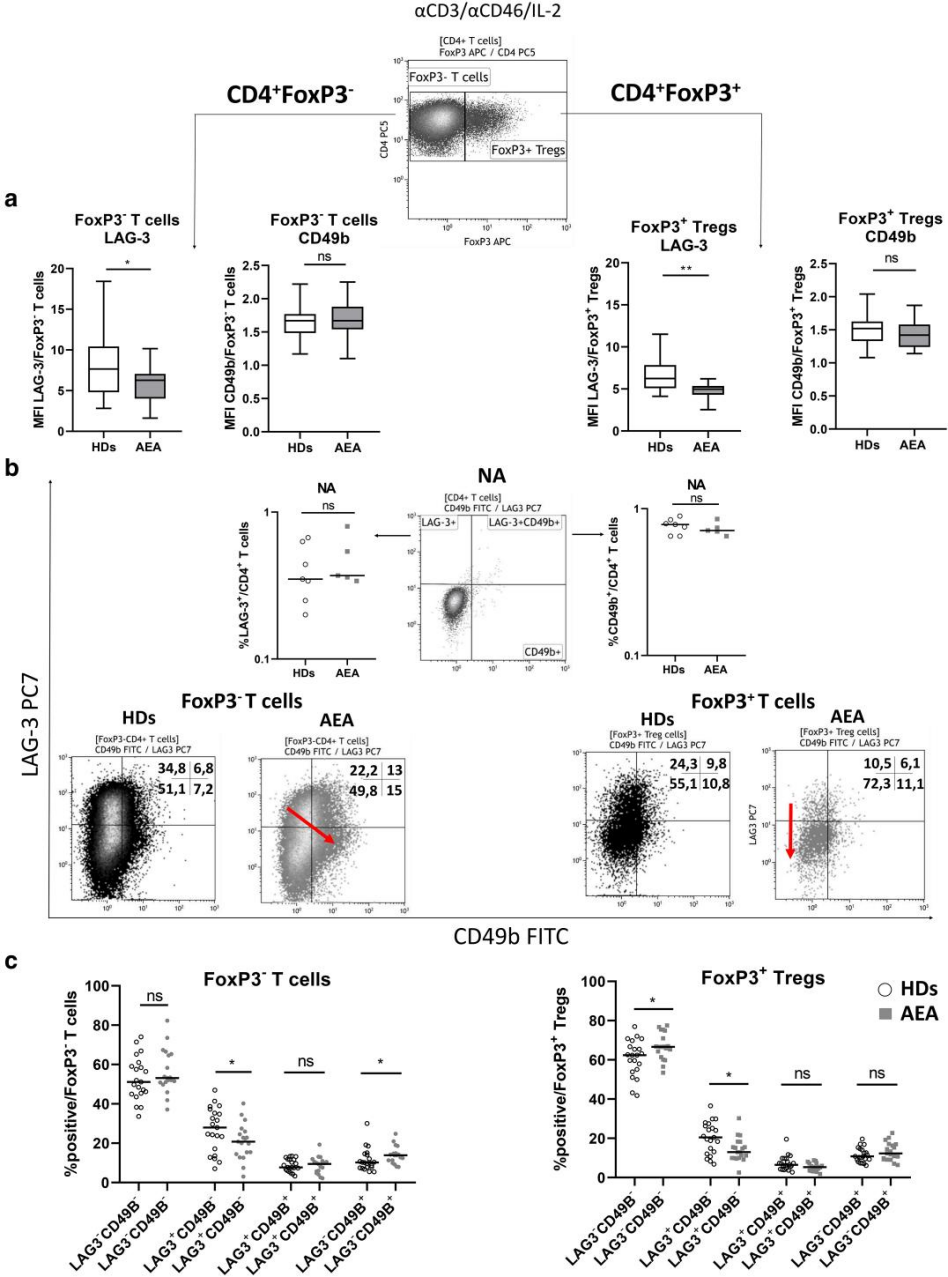
LAG-3 and CD49b expression on FoxP3^+^ and FoxP3^−^ CD4^+^ T cells activated via αCD3/αCD46/IL-2. (a) CD4^+^ T cells were categorized into two subsets based on FoxP3 expression: CD4 ^+^ FoxP3^−^ T cells and CD4^+^FoxP3^+^ Treg cells. CD4^+^FoxP3^−^ T cells from AEA patients exhibited reduced LAG-3 but normal CD49b expression (measured as median fluorescence intensity, MFI) compared to HDs. Identical results of decreased LAG-3 expression were observed within CD4^+^FoxP3^+^ Tregs in AEA patients. Data are presented as box & whiskers (median, min-max). (b) A representative dot plot demonstrates that nonactivated (NA) CD4^+^ T cells do not express LAG-3 and CD49b. Under NA condition, no differences in LAG-3 or CD49b expression were observed between a small group of HDs (n = 7) and AEA patients (n = 5). Following a division into CD4^+^FoxP3^−^ T cells and CD4 ^+^ FoxP3^+^ Treg cells, individual subsets of LAG-3^−^CD49b^−^, LAG-3^+^CD49b^−^, LAG-3^+^CD49b^+^ and LAG-3^−^CD49b^+^ T cells were analyzed within both HDs and AEA patients. Dot plots are representative of one healthy donor (1/19, black) and one patient with moderate AEA (1/20, grey). (c) Within CD4^+^FoxP3^+^ Tregs, the LAG-3^+^CD49b^−^ subset was diminished and compensated by elevated FoxP3^+^LAG-3^−^CD49b^−^ subset, however the CD4^+^FoxP3^−^ T cells showed a different pattern. Instead, diminished FoxP3^−^LAG-3^+^CD49b^−^ subset was compensated by elevated FoxP3^−^LAG-3^−^CD49b^+^ T cells in AEA patients when compared with HDs. Horizontal bars in graphs represent the median. Data were analyzed using a nonparametrical Mann–Whitney *U* test. ns (not significant), *P ≤ 0.05, **P ≤ 0.01, HDs, healthy donors; AEA, allergic eosinophilic asthma; FoxP3, Forkhead box 3; LAG-3, lymphocyte-activation gene-3.

AEA patients exhibited a normal frequency of CD4 ^+^ IL-10^+^ T cells and showed a trend towards increased IL-10 production per cell following αCD3/αCD46/IL-2 costimulation despite a reduced percentage of CD4^+^CD25^+^CD127^low^ Tregs in peripheral blood and decreased CD4^+^FoxP3^+^ T cell frequencies after *in vitro* stimulation (Supplementary Figure S6a). Thus, we next investigated whether CD4^+^ induced Tregs might be involved in increased IL-10 production. CD46 signaling strongly supports the generation of induced Tregs during the late phase of immune response, which are associated with upregulation of the surface markers LAG-3 and CD49b (Fig. 5) [21]. Therefore, we analyzed LAG-3 and CD49b expression separately within CD4 ^+^ FoxP3^−^ T cells and CD4^+^FoxP3^+^ Treg cells. Both subsets showed a decreased LAG-3, but normal CD49b expression in AEA patients (Fig. 5a). Identical results were observed when analyzed as a percentage of CD4^+^LAG-3^+^ and CD4^+^CD49b^+^ T cells (Supplementary Figure S6b). Next, we examined the distribution of CD4^+^ T cell subsets based on LAG-3 and CD49b following distribution into FoxP3^−^ and FoxP3^+^ T cells (Fig. 5b). We observed that within FoxP3^+^ Tregs, reduced LAG-3^+^ cells were substituted by increased FoxP3^+^LAG-3^−^CD49b^−^ population in AEA patients. Additionally, in the FoxP3^−^ T cell compartment, the decrease in LAG-3^+^ cells was compensated by an expansion of the FoxP3^−^LAG-3^−^CD49b^+^ subset in AEA patients.

### AEA patients show increased CD4^+^FoxP3^−^LAG-3^+^CD49b^+^ T cells and CD4^+^FoxP3^−^LAG-3^−^CD49b^+^ T cells after stimulation *in vitro*

**Figure 6:**
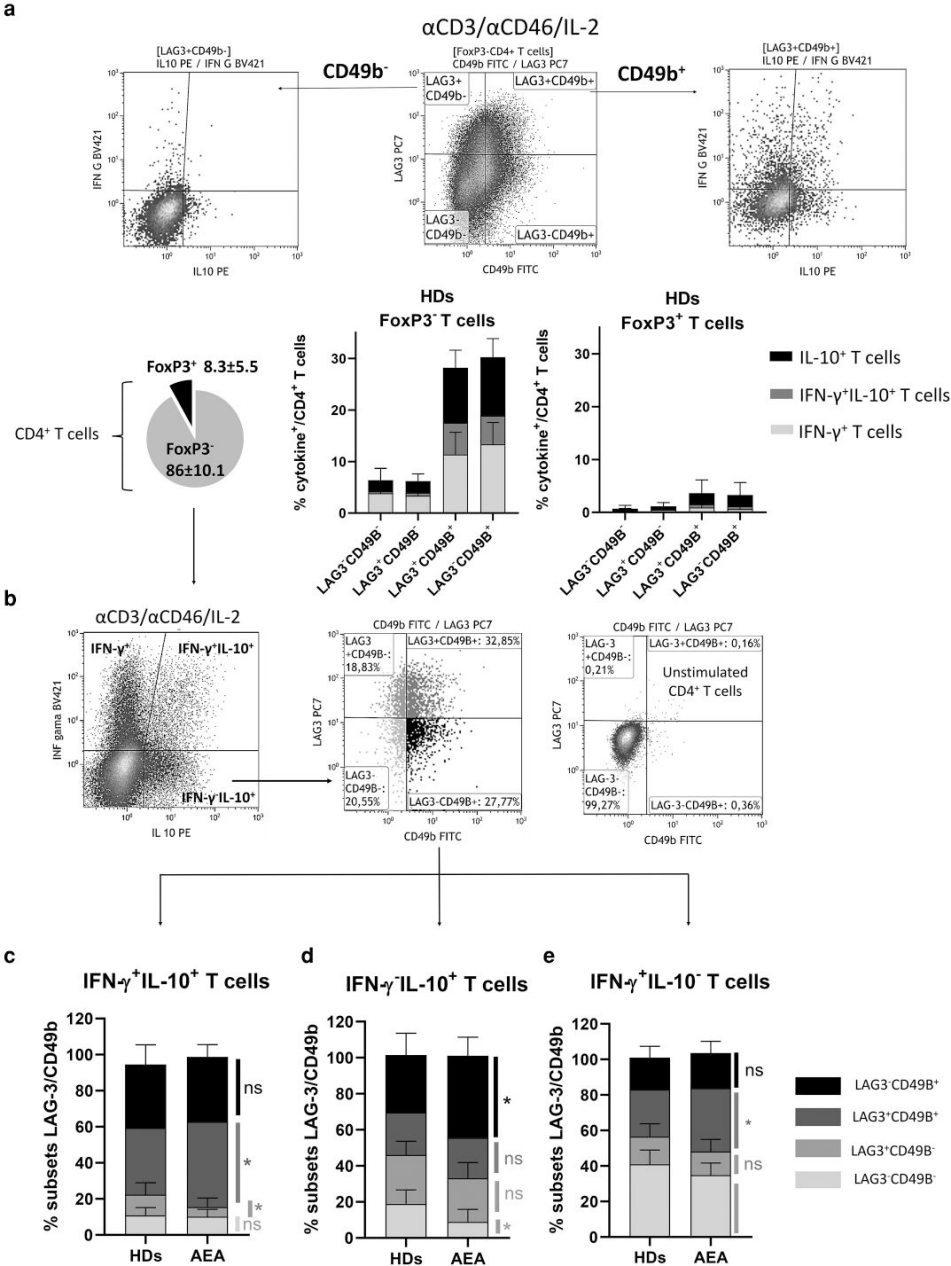
LAG-3/CD49b subsets in relation to IFN-γ and IL-10 production following αCD3/αCD46/IL-2 activation *in vitro.* (a) Based on division into subsets according to the LAG-3 and CD49b expression, CD4^+^FoxP3^−^ T cells and CD4^+^FoxP3^+^ Treg cells from HDs were further analyzed in the context of IFN-γ and IL-10 production. It was shown that dominant cytokine producers within both FoxP3^−^CD4^+^ T cells and CD4^+^FoxP3^+^ Treg cells are subsets expressing CD49b, although CD4^+^FoxP3^+^ Treg cells represented a substantially smaller fraction of CD4^+^ T cells (8.3 ± 5.5%). (b) Next, FoxP3^−^CD4^+^ T cells were divided into four subsets based on IFN-γ and IL-10 production (IFN-γ^−^IL-10^−^, IFN-γ^+^IL-10^−^, IFN-γ^+^IL-10^+^, and IFN-γ^−^IL-10^+^) followed by division of each subset according to LAG-3 and CD49b expression. Dot plots are representative of one healthy donor and show cytokine production in activated CD4^+^FoxP3^−^ T cells (αCD3/αCD46/IL-2), LAG-3/CD49b expression within activated CD4^+^FoxP3^−^IFN-γ^−^IL-10^+^ T cells and nonstimulated T cells, respectively. (c) We observed that within the subset producing both cytokines IFN-γ and IL-10, AEA patients exhibited an upregulated population with a CD4^+^IFN-γ^+^IL-10^+^LAG-3^+^CD49b^+^ phenotype, which has been described previously in Tr1 cells. In the IFN-γ^−^IL-10^+^ subset, AEA patients showed an increased proportion of T cells with a CD4^+^IFN-γ^−^IL-10^+^LAG-3^−^CD49b^+^ phenotype, which have been previously described to be associated with nonclassical CD49b^+^ regulatory T cells. Interestingly, CD4^+^IFN-γ^+^ T cells in AEA patients also showed a higher proportion of a subset expressing both LAG-3 and CD49b, which are markers associated with regulatory T cells. The data are presented as vertical stacked bars that depict mean + SD highlighted in different shades of grey for each subset according to LAG-3 and CD49b expression. Data were analyzed using a nonparametrical Mann–Whitney *U* test. ns (nyot significant), *P ≤ 0.05, **P ≤ 0.01, HDs, healthy donors; AEA, allergic eosinophilic asthma; LAG-3, lymphocyte-activation gene-3; SD, standard deviation.

Next, we investigated differences among CD4^+^ T cell subsets defined by LAG-3 and CD49b expression, with a focus on their functional characteristics, particularly the production of IFN-γ and IL-10 (Fig. 6). We observed that the major producers of both cytokines were the FoxP3^−^CD49b^+^ and FoxP3^+^CD49b^+^ T cells. However, since FoxP3^+^ T cells represent a substantially smaller fraction of circulating CD4^+^ T cells (8.3 ± 5.5%), FoxP3^−^ T cells, which represent the majority of CD4^+^ T cell compartment (86.0 ± 10.1%), predominate in overall cytokine secretion (Fig. 6a). Next, we focused on IFN-γ^+^, IFN-γ^+^ IL-10^+^, and IL-10^+^ cells and analyzed LAG-3 and CD49b expression within those subsets (Fig. 6b). We found that within IFN-γ^+^IL-10^+^ T cells, there was an expanded subset of T cells exhibiting FoxP3^−^LAG-3^+^CD49b^+^ phenotype in AEA patients (*P* = 0.046; 47.4 ± 9.4% AEA vs 37.1 ± 10.1% HDs), that has been previously described to be associated with induced Tr1Tregs (Fig. 6c). Additionally, within IL-10^+^ T cells, we found an increased percentage of cells expressing only CD49b (*P* = 0.037; 41.5 ± 10% AEA vs 31.7 ± 12% HDs) in AEA patients (Fig. 6d). Eventually, LAG-3^+^CD49b^+^ subset was also increased within IFN-γ^+^ Th1 cells in AEA patients (*P* = 0.046; 35.5 ± 8.6% AEA vs 26.5 ± 9.7% HDs), possibly indicating an initial stage towards complosome-driven induced Tregs reprogramming (Fig. 6e). These findings suggest that, although AEA patients exhibit a decreased FoxP3^+^ Treg cell compartment, inducible Tregs subsets characterized by FoxP3^−^IL-10^+^LAG-3^+^CD49b^+^ and FoxP3^−^IL-10^+^LAG-3^−^CD49b^+^ phenotype may functionally compensate FoxP3^+^ T cells by more pronounced differentiation and enhanced IL-10 production.

## Discussion

In this study, we focused on the regulatory phase of CD4^+^ T cell responses in AEA, with particular emphasis on complosome-dependent CD46 signaling and the transition from Th1 cells toward IL-10-producing induced Tregs. By combining *ex vivo* phenotypic analysis with a mechanistic αCD3/αCD46/IL-2 activation model, we demonstrate that despite a reduced FoxP3^+^ Treg compartment and enhanced Th1 activity, CD4^+^ T cells from patients with controlled AEA retain the capacity to induce IL-10 production upon CD46-mediated activation. These findings indicate that alterations in CD46/C3b dynamics and cytokine output shape the balance between IFN-γ–driven effector responses and IL-10–mediated regulation, and suggest that inducible regulatory mechanisms may partially compensate for impaired classical Treg-mediated control in AEA [34, 35].

Our *ex vivo* analyses indicate a chronically primed effector T cell environment in controlled AEA, characterized by elevated PD1 expression, reduced FoxP3^+^ Treg cells, increased memory CD4^+^ T cells, and increased plasma IFN-γ. These observations are consistent with previous reports describing impaired Tregs compartment [6, 36, 37] together with enhanced effector activation in asthma [12, 38, 39]. Collectively, these features suggest that even in clinically stable disease, CD4^+^ T cells in AEA remain in a state of sustained immune activation.

Interpretation of regulatory pathways in asthma is further complicated by pronounced seasonal variability. IL-10 levels assessed in adult patients with AEA have shown heterogeneous results [7, 29, 30] and are rarely interpreted in the context of the pollen season [40]. However, several studies have demonstrated seasonal changes in cytokine responses and airway inflammation in sensitized individuals, with more pronounced T2-driven activation during periods of allergen exposure [41–43]. Sampling outside the pollen season was undertaken to minimize T2-driven seasonal effects and to assess more stable features of CD4^+^ T cell phenotype and complosome-driven activation.

Within the αCD3/αCD46/IL-2 activation model, CD4^+^ T cells from patients with controlled AEA exhibited a pronounced T1 response, characterized by increased IFN-γ production compared with HDs. Enhanced T1 activity has been reported in autoimmune diseases such as systemic lupus erythematosus and rheumatoid arthritis; however, in these conditions, it is typically associated with reduced CD46 expression and impaired CD46-dependent IL-10 induction [15, 44, 45]. In contrast, CD4^+^ T cells from AEA patients displayed increased surface CD46 expression together with altered C3b deposition, while retaining the capacity to produce IL-10 upon CD46-mediated activation. These findings suggest a distinct pattern of complosome regulation in AEA, in which enhanced Th1 activity coexists with preserved CD46-dependent regulatory competence during the late phase of T cell activation [15, 20, 44–46].

In addition, we observed that memory CD4^+^ T cells in AEA exhibited higher surface expression of CD46 compared with their naive counterparts, consistent with previous reports describing differential CD46 expression across human CD4^+^ T cell subsets [19, 28]. Although increased CD46 expression does not necessarily translate into enhanced signalling at the single-cell level, the predominance of memory-phenotype CD4^+^ T cells in AEA may influence complosome-associated regulatory pathways under conditions of immune activation. In the context of increased IFN-γ production and preserved CD46-dependent IL-10 competence, this memory CD4^+^ T cell compartment may contribute to the distinct pattern of complosome regulation observed in controlled AEA.

Despite increased IFN-γ production, αCD3/αCD46/IL-2-induced IL-10 secretion remained comparable between HDs and AEA patients. Nevertheless, IL-10 secretion was not reflected in increased frequency of CD4^+^IL-10^+^ T cells, but rather in increased IL-10 production at the single-cell level. This was clearly demonstrated by the ELISpot assay, where AEA patients showed no difference in the number of IL-10^+^ T cells, yet they had significantly larger IL-10 spot size, indicating increased IL-10 secretion per cell. The interpretation of spot size as an indicator of cytokine production is supported by other studies [47, 48], where spot size and intensity reflected the amount of cytokine released by individual cells rather than cell frequency alone. Consistent with these findings, intracellular flow cytometry revealed increased MFI for IL-10 in CD4^+^ T cells from AEA patients, further supporting an enhanced cytokine production per cell. Taken together, these results indicate that IL-10 regulatory ability in controlled AEA is maintained not by increased numbers of CD4^+^IL-10^+^ T cells, but by stronger cytokine production at the single-cell level. Furthermore, previous studies showed that CD46 co-stimulation induced IL-10 predominantly in CD4^+^FoxP3^−^ T cells named as type 1 Tregs [15, 19, 20]. Our findings extended this work by showing that CD46-dependent IL-10 induction remains functional despite reduced FoxP3^+^ Treg cells in AEA patients.

We further observed that IL-10-producing CD4^+^ T cells were predominantly enriched within the FoxP3^−^CD49b^+^ compartment, and that patients with controlled AEA showed an overall shift toward CD49b^+^ subsets among cytokine-producing CD4^+^ T cells. Importantly, this pattern was not due to low frequencies of LAG-3^+^ or CD49b^+^ cells, but rather reflected the overall low frequency of FoxP3^+^IL-10^+^ T cells, which remained in low numbers even after *in vitro* stimulation. Reduced surface expression of LAG-3 may also reflect activation-induced release [49, 50], although this remains to be verified in future studies. Given that CD49b is associated with activation-induced IL-10 production and that LAG-3/CD49b expression reflects an activation-associated rather than lineage-defining phenotype, these findings support an activation-driven regulatory state rather than stable Tr1 differentiation. Together, these data suggest that CD46-dependent activation, likely shaped by altered C3b dynamics, imprints a transient regulatory signature on IL-10-producing CD4^+^FoxP3^−^ T cells in controlled AEA.

Taken together, our findings help to place previously disparate observations into a coherent framework. CD46-driven IL-10 induction has been well characterized in mechanistic studies [17, 18, 29, 51], but it has not been examined in AEA. Conversely, studies that focused on Tregs in asthma consistently report reduced FoxP3^+^ Treg cells and variable IL-10 levels [7, 36, 37, 52] without linking these abnormalities to CD46/complosome signaling and function. *Ex vivo* observed changes in CD4^+^ T cell phenotype coupled with activation-induced CD46 functional response indicate that the capacity of CD4^+^FoxP3^−^ T cells to upregulate CD46-dependent IL-10 secretion upon αCD3/αCD46/IL-2 stimulation may represent an activation-associated mechanism that could help compensate reduced FoxP3^+^ Treg cells in AEA.

This may have potential clinical implications. The preservation of activation-induced IL-10 production despite enhanced IFN-γ responses highlights the CD46-dependent pathway as a potential candidate for supporting regulatory immune responses, with implications that may extend beyond AEA. In this context, altered CD46/C3b dynamics, together with a shift toward activated CD4^+^ T cell subsets expressing CD49b, may reflect features of dysregulated CD4^+^ T cell activation in AEA. These observations further suggest the possibility that modulation of complosomal signalling or CD46-dependent IL-10 induction could represent an interesting avenue for future immunomodulatory approaches not only in chronic allergic inflammation.

However, this study has several limitations. The αCD3/αCD46/IL-2 activation model is reductionist and does not reflect allergen properties or the complexity of airway-resident CD4^+^ T cells and specific microenvironment present in airway tissue. The modest cohort size and restriction to out-of-season sampling of adult AEA patients may limit applicability across different asthma phenotypes and age (children vs. adults). Further, LAG-3 and CD49b are informative activation-associated markers but cannot define stable regulatory CD4^+^ T cell lineages *in vivo*. Future work incorporating allergen stimuli and airway sampling will be essential to clarify how complosome and CD46-driven IL-10 competence operates *in vivo* and how it influences the clinical manifestation of the disease.

## Conclusion

In conclusion, our data indicate that controlled AEA is associated with a persistent imbalance in CD4^+^ T cell regulation, characterized by enhanced T1 activity, altered complosome-associated CD46 signalling and reduced frequencies of FoxP3^+^ Tregs. Using *ex vivo* phenotypic analyses and a defined CD46-dependent activation model, we show that CD4^+^ T cells retain the ability to mount activation-induced IL-10 responses predominantly within the FoxP3^−^ compartment, despite diminished classical Treg representation. Importantly, these findings reflect inducible regulatory capacity revealed under experimental activation conditions rather than direct evidence of ongoing regulatory activity *in vivo*. Altered CD46/C3b dynamics and enrichment of CD49b-expressing IL-10-producing CD4^+^ T cells highlight complosome-related features of T cell activation that may be informative for future studies addressing immune regulation and therapeutic modulation in AEA.

## Supplementary Material

uxag017_Supplementary_Data

## Data Availability

The data underlying this article are available from the corresponding author on reasonable request.

## Abbreviations:

AEA: allergic eosinophilic asthma
αCD: anti-CD
ECP: eosinophilic cationic protein
FoxP3: forkhead box 3
HDs: healthy donors
LAG-3: lymphocyte activation gene-3
MCP: membrane cofactor protein (CD46)
NA: nonactivated
sCD46: soluble CD46
sIL-2RA: soluble IL-2 α chain
SNs: supernatants
Tr1: type 1 regulatory T cell
Treg: regulatory T cell

## References

[1] Boonpiyathad T, Sözener ZC, Satitsuksanoa P, Akdis CA. Immunologic mechanisms in asthma. Semin Immunol 2019, 46, 101333. 31703832 10.1016/j.smim.2019.101333

[2] Froidure A, Vandenplas O, D’Alpaos V, Evrard G, Pilette C. Persistence of asthma following allergen avoidance is associated with proTh2 myeloid dendritic cell activation. Thorax 2015, 70, 967–73. 26103997 10.1136/thoraxjnl-2014-206364

[3] Gray NJ, Frew AJ. Allergen avoidance in asthma: is there a role? Curr Treat Options Allergy 2014, 1, 186–97.

[4] Ray A, Khare A, Krishnamoorthy N, Qi Z, Ray P. Regulatory T cells in many flavors control asthma. Mucosal Immunol 2010, 3, 216–29. 20164832 10.1038/mi.2010.4 PMC3039023

[5] Muehling LM, Lawrence MG, Woodfolk JA. Pathogenic CD4+ T cells in patients with asthma. J Allergy Clin Immunol 2017, 140, 1523–40. 28442213 10.1016/j.jaci.2017.02.025 PMC5651193

[6] Shi Y, Wan H-y, AI X-y, Zhu H-x, Tang W, Ma J-y, et al An increased ratio of Th2/treg cells in patients with moderate to severe asthma. Chin Med J 2013, 126, 2248–53. 23786933

[7] Xu Y-Q, Gao Y-D, Yang J, Guo W. A defect of CD4 + CD25+ regulatory T cells in inducing interleukin-10 production from CD4+ T cells under CD46 costimulation in asthma patients. J Asthma 2010, 47, 367–73. 20528588 10.3109/02770903.2010.481340

[8] Kraszula Ł, Eusebio M-O, Kuna P, Pietruczuk M. Relationship between CCR5+FoxP3+ treg cells and forced expiratory volume in 1s, peak expiratory flow in patients with severe asthma. Postepy Dermatol Alergol 2021, 38, 262–8. 34408594 10.5114/ada.2021.106202 PMC8362744

[9] Gavett SH, O'Hearn DJ, Li X, Huang SK, Finkelman FD, Wills-Karp M. Interleukin 12 inhibits antigen-induced airway hyperresponsiveness, inflammation, and Th2 cytokine expression in mice. J Exp Med 1995, 182, 1527–36. 7595222 10.1084/jem.182.5.1527 PMC2192202

[10] Hansen G, McIntire JJ, Yeung VP, Berry G, Thorbecke GJ, Chen L, et al CD4^+^ T helper cells engineered to produce latent TGF-β1 reverse allergen-induced airway hyperreactivity and inflammation. J Clin Invest 2000, 105, 61–70. 10619862 10.1172/JCI7589 PMC382583

[11] Shannon J, Ernst P, Yamauchi Y, Olivenstein R, Lemiere C, Foley S, et al Differences in airway cytokine profile in severe asthma compared to moderate asthma. Chest 2008, 133, 420–6. 18071017 10.1378/chest.07-1881

[12] Wisniewski JA, Muehling LM, Eccles JD, Capaldo BJ, Agrawal R, Shirley D-A, et al TH1 signatures are present in the lower airways of children with severe asthma, regardless of allergic status. J Allergy Clin Immunol 2018, 141, 2048–2060.e13. 28939412 10.1016/j.jaci.2017.08.020 PMC5860937

[13] Kuo C-HS, Pavlidis S, Loza M, Baribaud F, Rowe A, Pandis I, et al T-helper cell type 2 (Th2) and non-Th2 molecular phenotypes of asthma using sputum transcriptomics in U-BIOPRED. Eur Respir J 2017, 49, 1602135. 28179442 10.1183/13993003.02135-2016

[14] Freeley S, Kemper C, Le Friec G. The ‘ins and outs’ of complement-driven immune responses. Immunol Rev 2016, 274, 16–32. 27782335 10.1111/imr.12472 PMC5102160

[15] Cardone J, Le Friec G, Vantourout P, Roberts A, Fuchs A, Jackson I, et al Complement regulator CD46 temporally regulates cytokine production by conventional and unconventional T cells. Nat Immunol 2010, 11, 862–71. 20694009 10.1038/ni.1917 PMC4011020

[16] West EE, Kemper C. Complosome—the intracellular complement system. Nat Rev Nephrol 2023, 19, 426–39. 37055581 10.1038/s41581-023-00704-1 PMC10100629

[17] Le Friec G, Sheppard D, Whiteman P, Karsten CM, Shamoun SA-T, Laing A, et al The CD46-jagged1 interaction is critical for human TH1 immunity. Nat Immunol 2012, 13, 1213–21. 23086448 10.1038/ni.2454 PMC3505834

[18] Liszewski MK, Kolev M, Le Friec G, Leung M, Bertram PG, Fara AF, et al Intracellular complement activation sustains T cell homeostasis and mediates effector differentiation. Immunity 2013, 39, 1143–57. 24315997 10.1016/j.immuni.2013.10.018 PMC3865363

[19] Ni Choileain S, Hay J, Thomas J, Williams A, Vermeren MM, Benezech C, et al TCR-stimulated changes in cell surface CD46 expression generate type 1 regulatory T cells. Sci Signal 2017, 10, eaah6163. 29066539 10.1126/scisignal.aah6163

[20] Kemper C, Chan AC, Green JM, Brett KA, Murphy KM, Atkinson JP. Activation of human CD4+ cells with CD3 and CD46 induces a T-regulatory cell 1 phenotype. Nature 2003, 421, 388–92. 12540904 10.1038/nature01315

[21] Gagliani N, Magnani CF, Huber S, Gianolini ME, Pala M, Licona-Limon P, et al Coexpression of CD49b and LAG-3 identifies human and mouse T regulatory type 1 cells. Nat Med 2013, 19, 739–46. 23624599 10.1038/nm.3179

[22] Huang W, Solouki S, Carter C, Zheng S-G, August A. Beyond type 1 regulatory T cells: co-expression of LAG3 and CD49b in IL-10-producing T cell lineages. Front Immunol 2018, 9, 2625. 30510554 10.3389/fimmu.2018.02625 PMC6252342

[23] Ni Choileain S, Weyand NJ, Neumann C, Thomas J, So M, Astier AL. The dynamic processing of CD46 intracellular domains provides a molecular rheostat for T cell activation. PLoS One 2011, 6, e16287. 21283821 10.1371/journal.pone.0016287 PMC3023775

[24] Liszewski MK, Post TW, Atkinson JP. Membrane cofactor protein (MCP or CD46): newest member of the regulators of complement activation gene cluster. Annu Rev Immunol 1991, 9, 431–55. 1910685 10.1146/annurev.iy.09.040191.002243

[25] Vieira PL, Christensen JR, Minaee S, O’Neill EJ, Barrat FJ, Boonstra A, et al IL-10-secreting regulatory T cells do not express Foxp3 but have comparable regulatory function to naturally occurring CD4+CD25+ regulatory T cells 1. J Immunol 2004, 172, 5986–93. 15128781 10.4049/jimmunol.172.10.5986

[26] Ni Choileain S, Astier AL. CD46 processing: a means of expression. Immunobiology 2012, 217, 169–75. 21742405 10.1016/j.imbio.2011.06.003 PMC4363545

[27] Kickler K, Choileain SN, Williams A, Richards A, Astier AL. Calcitriol modulates the CD46 pathway in T cells. PLoS One 2012, 7, e48486. 23144765 10.1371/journal.pone.0048486 PMC3483209

[28] Christmas SE, De La Mata Espinosa CT, Halliday D, Buxton CA, Cummerson JA, Johnson PM. Levels of expression of complement regulatory proteins CD46, CD55 and CD59 on resting and activated human peripheral blood leucocytes. Immunology 2006, 119, 522–8. 16999828 10.1111/j.1365-2567.2006.02467.x PMC2265819

[29] Tsai Y-G, Niu D-M, Yang KD, Hung C-H, Yeh Y-J, Lee C-Y, et al Functional defects of CD46-induced regulatory T cells to suppress airway inflammation in mite allergic asthma. Lab Invest 2012, 92, 1260–9. 22751347 10.1038/labinvest.2012.86

[30] Melgaard ME, Jensen SK, Eliasen A, Pedersen C-ET, Thorsen J, Mikkelsen M, et al Asthma development is associated with low mucosal IL-10 during viral infections in early life. Allergy 2024, 79, 2981–92. 39221476 10.1111/all.16276

[31] Gupta A, Dimeloe S, Richards DF, Chambers ES, Black C, Urry Z, et al Defective IL-10 expression and in vitro steroid-induced IL-17A in paediatric severe therapy-resistant asthma. Thorax 2014, 69, 508–15. 24347461 10.1136/thoraxjnl-2013-203421

[32] Reddel HK, Bacharier LB, Bateman ED, Brightling CE, Brusselle GG, Buhl R, et al Global initiative for asthma strategy 2021: executive summary and rationale for key changes. Am J Respir Crit Care Med 2022, 205, 17–35. 34658302 10.1164/rccm.202109-2205PP PMC8865583

[33] Kreher CR, Dittrich MT, Guerkov R, Boehm BO, Tary-Lehmann M. CD4+ and CD8+ cells in cryopreserved human PBMC maintain full functionality in cytokine ELISPOT assays. J Immunol Methods 2003, 278, 79–93. 12957398 10.1016/s0022-1759(03)00226-6

[34] D’Aiuto V, Mormile I, Granata F, Romano A, Della Casa F, Mignogna G, et al Eosinophil-driven vs. Eosinophil-associated severe asthma: practical implications for target treatment. Int J Mol Sci 2025, 26, 1729. 40004192 10.3390/ijms26041729 PMC11855446

[35] Gans MD, Gavrilova T. Understanding the immunology of asthma: pathophysiology, biomarkers, and treatments for asthma endotypes. Paediatr Respir Rev 2020, 36, 118–27. 31678040 10.1016/j.prrv.2019.08.002

[36] Provoost S, Maes T, Van Durme YM, Gevaert P, Bachert C, Schmidt-Weber CB, et al Decreased FOXP3 protein expression in patients with asthma. Allergy 2009, 64, 1539–46. 19392991 10.1111/j.1398-9995.2009.02056.x

[37] Li C, Sheng A, Jia X, Zeng Z, Zhang X, Zhao W, et al Th17/treg dysregulation in allergic asthmatic children is associated with elevated notch expression. J Asthma 2018, 55, 1–7. 28463581 10.1080/02770903.2016.1266494

[38] Jie X, Wang D, Da H, Li H, Zhao H, He J, et al Increased inhibitory surface marker PD-1 expression in CD4+T cells and Th2+T cells in allergen-specific immunotherapy. Immunobiology 2024, 229, 152824. 38875763 10.1016/j.imbio.2024.152824

[39] Huang F, Yin J-N, Wang H-B, Liu S-Y, Li Y-N. Association of imbalance of effector T cells and regulatory cells with the severity of asthma and allergic rhinitis in children. Allergy Asthma Proc 2017, 38, 70–7. 10.2500/aap.2017.38.4076 29046188

[40] Ceyhan BB, Enc FY, Sahin S. IL-2 and IL-10 levels in induced sputum and serum samples of asthmatics. J Investig Allergol Clin Immunol 2004, 14, 80–5. 15160446

[41] Panzner P, Malkusová I, Vachová M, Liška M, Brodská P, Růžičková O, et al Bronchial inflammation in seasonal allergic rhinitis with or without asthma in relation to natural exposure to pollen allergens. Allergol Immunopathol (Madr) 2015, 43, 3–9. 24075536 10.1016/j.aller.2013.06.009

[42] Wosinska-Becler K, Plewako H, Håkansson L, Rak S. Cytokine production in peripheral blood cells during and outside the pollen season in birch-allergic patients and non-allergic controls. Clin Exp Allergy 2004, 34, 123–30. 14720272 10.1111/j.1365-2222.2004.01850.x

[43] Baraldi E, Carrà S, Dario C, Azzolin N, Ongaro R, Marcer G, et al Effect of natural grass pollen exposure on exhaled nitric oxide in asthmatic children. Am J Respir Crit Care Med 1999, 159, 262–6. 9872848 10.1164/ajrccm.159.1.9804063

[44] West EE, Kemper C. Complement and T cell metabolism: food for thought. Immunometabolism 2019, 1, e190006. 31328019 10.20900/immunometab20190006 PMC6642051

[45] Kolev M, Dimeloe S, Le Friec G, Navarini A, Arbore G, Povoleri GA, et al Complement regulates nutrient influx and metabolic reprogramming during Th1 cell responses. Immunity 2015, 42, 1033–47. 26084023 10.1016/j.immuni.2015.05.024 PMC4518498

[46] Arbore G, Kemper C. A novel ‘complement-metabolism-inflammasome axis’ as a key regulator of immune cell effector function. Eur J Immunol 2016, 46, 1563–73. 27184294 10.1002/eji.201546131 PMC5025719

[47] Maeda C, Iizuka A, Miyata H, Kondou R, Ashizawa T, Kanematsu A, et al Alternative evaluation of an ELISPOT assay using cytokine activity as a novel parameter. Anticancer Res 2021, 41, 3825–31. 34281842 10.21873/anticanres.15175

[48] Sibley LS, White AD, Marriott A, Dennis MJ, Williams A, Marsh PD, et al ELISPOT refinement using spot morphology for assessing host responses to Tuberculosis. Cells 2012, 1, 5–14. 24710359 10.3390/cells1010005 PMC3972643

[49] Workman CJ, Cauley LS, Kim I-J, Blackman MA, Woodland DL, Vignali DAA. Lymphocyte activation gene-3 (CD223) regulates the size of the expanding T cell population following antigen activation in vivo. J Immunol Baltim 2004, 172, 5450–5. 10.4049/jimmunol.172.9.5450 15100286

[50] Li N, Wang Y, Forbes K, Vignali KM, Heale BS, Saftig P, et al Metalloproteases regulate T-cell proliferation and effector function via LAG-3. Embo J 2007, 26, 494–504. 17245433 10.1038/sj.emboj.7601520 PMC1783452

[51] Astier AL, Meiffren G, Freeman S, Hafler DA. Alterations in CD46-mediated Tr1 regulatory T cells in patients with multiple sclerosis. J Clin Invest 2006, 116, 3252–7. 17099776 10.1172/JCI29251 PMC1635165

[52] Hartl D, Koller B, Mehlhorn AT, Reinhardt D, Nicolai T, Schendel DJ, et al Quantitative and functional impairment of pulmonary CD4 + CD25hi regulatory T cells in pediatric asthma. J Allergy Clin Immunol 2007, 119, 1258–66. 17412402 10.1016/j.jaci.2007.02.023

