# Supplementary material for: Reduced IL-10 production by FoxP3+IL-10+ Treg cells is partially compensated by complosome-associated induction of regulatory FoxP3−IL-10+ T cells in allergic eosinophilic asthma
Source: Clin Exp Immunol. 2026 Mar 25;220(1):uxag017. doi: 10.1093/cei/uxag017 (PMC13064513; doi:10.1093/cei/uxag017)
Supplement: uxag017_Supplementary_Data [file uxag017_supplementary_data.zip › Supplementary Material .docx]

**Supplementary material**

**Figure S1: Peripheral blood eosinophils, ECP and specific IgE**

**
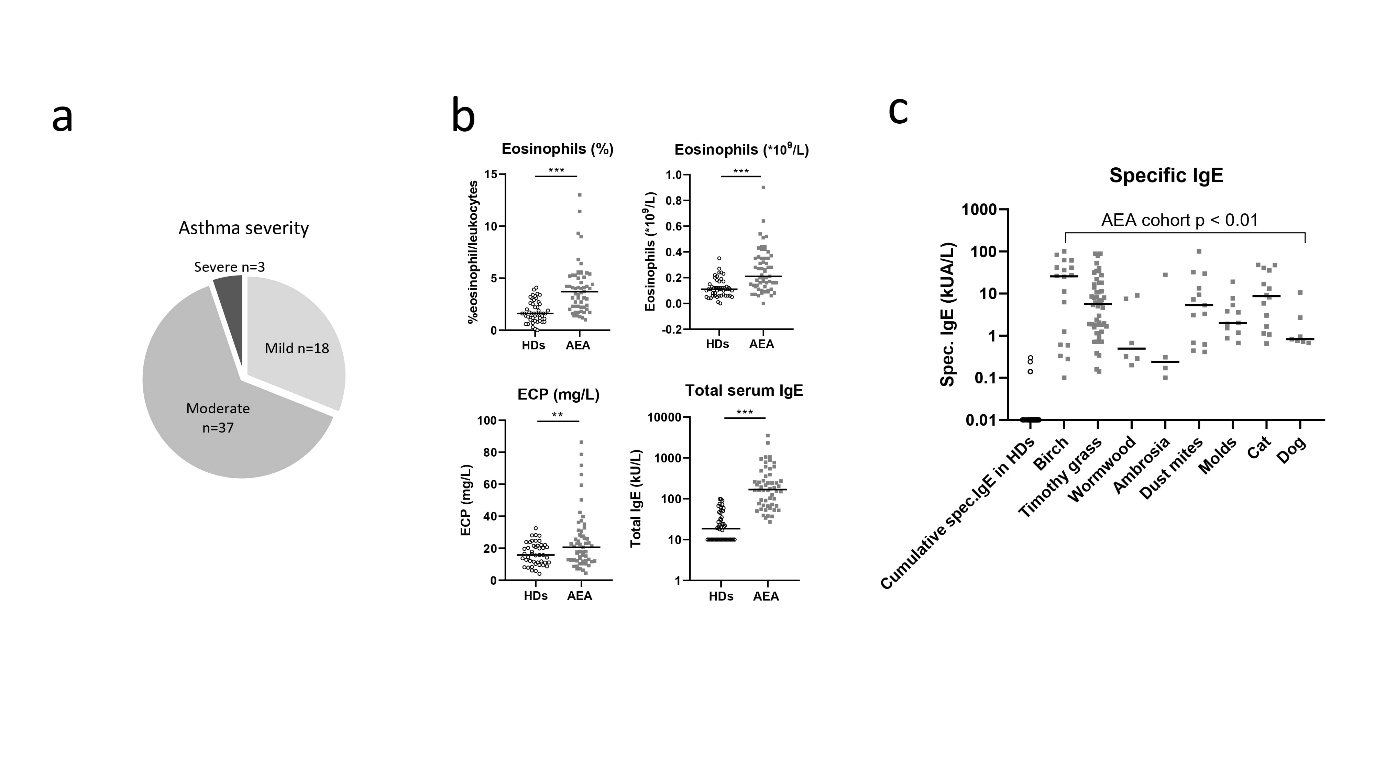
**

(a) A representative pie chart shows our patient cohort with compensated AEA with a stratification based on asthma severity (mild, moderate, severe) according to the GINA guidelines. (b) The relative (%) and absolute counts of eosinophils in peripheral blood were increased in AEA patients, as well as serum concentration of eosinophilic cationic protein (ECP) and total serum levels of IgE. Data were analyzed using an unpaired T-test. (c) Our AEA cohort showed significantly elevated concentrations of specific IgE in serum, especially against birch or timothy grass alone or in combination with other seasonal or perennial allergens (dust mites, molds, pets). Data were evaluated using the Kruskall-Wallis test with Dunn’s correction. The horizontal bar in graphs (b) and (c) represents the median. **p ≤ 0.01, ***p ≤ 0.0001. AEA, allergic eosinophilic asthma; GINA, Global Initiative for Asthma.

**Figure S2: Gating strategy used for CD4^+^ T cell phenotyping**

**
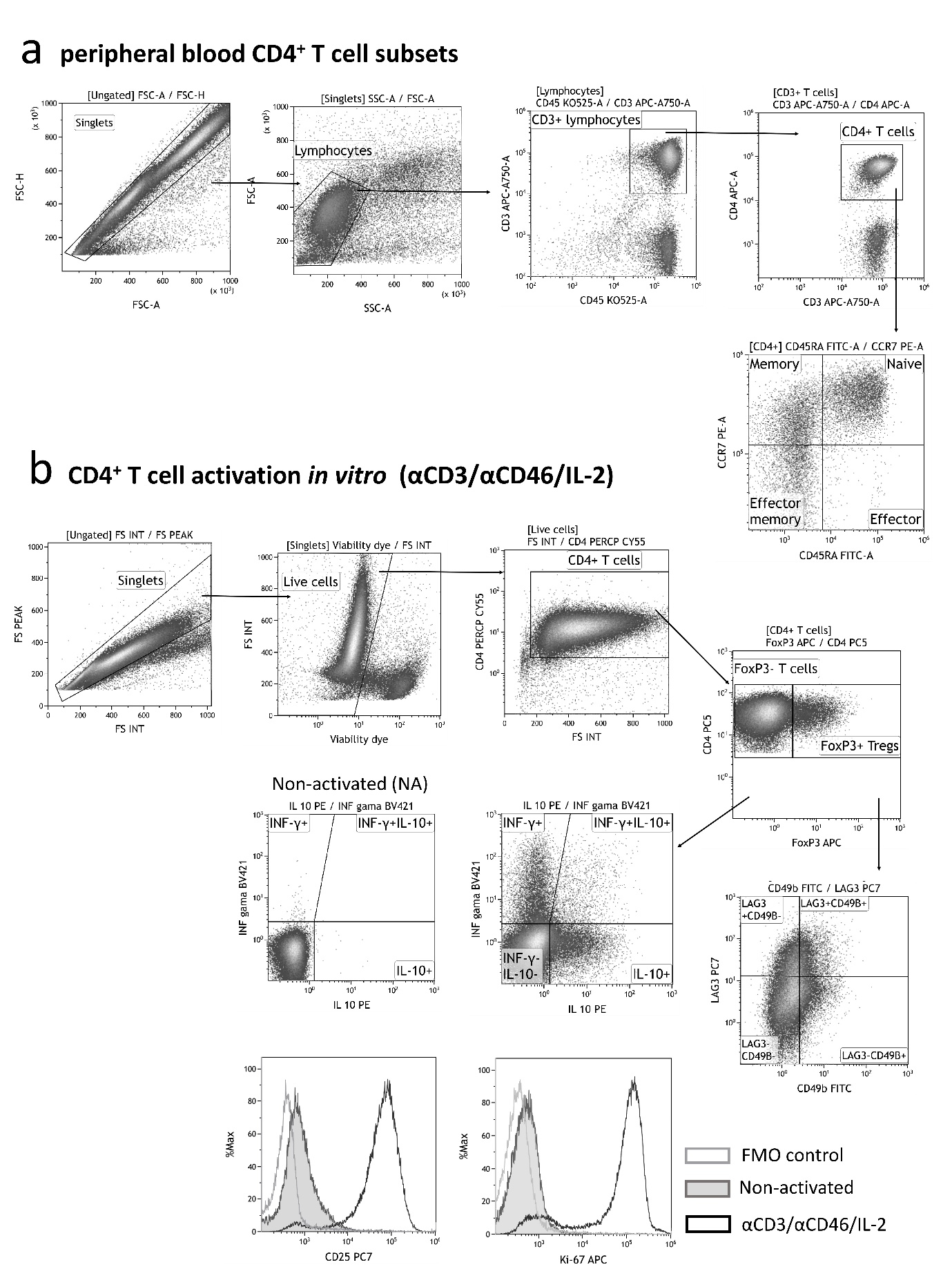
**

Peripheral blood CD4^+^ T cell subsets were analyzed based on the gating strategy described in section (a) using a DX flex flow cytometer (*Beckman Coulter, Brea, CA, USA)*. CD4^+^ T cells, isolated from PBMC by magnetic separation and subsequently activated *in vitro,* were analyzed following the gating strategy outlined in section (b) with a Navios EX 3L10C flow cytometer (*Beckman Coulter, Brea, CA, USA).*The dot plots are representative of one healthy donor from n=49, and were generated using Kaluza analysis software (*Beckman Coulter, Brea, CA, USA)*.

**Figure S3: ELISpot and IFN-γ and IL-10 expression within CD4^+^FoxP3^+^ and CD4^+^FoxP3^-^ T cells
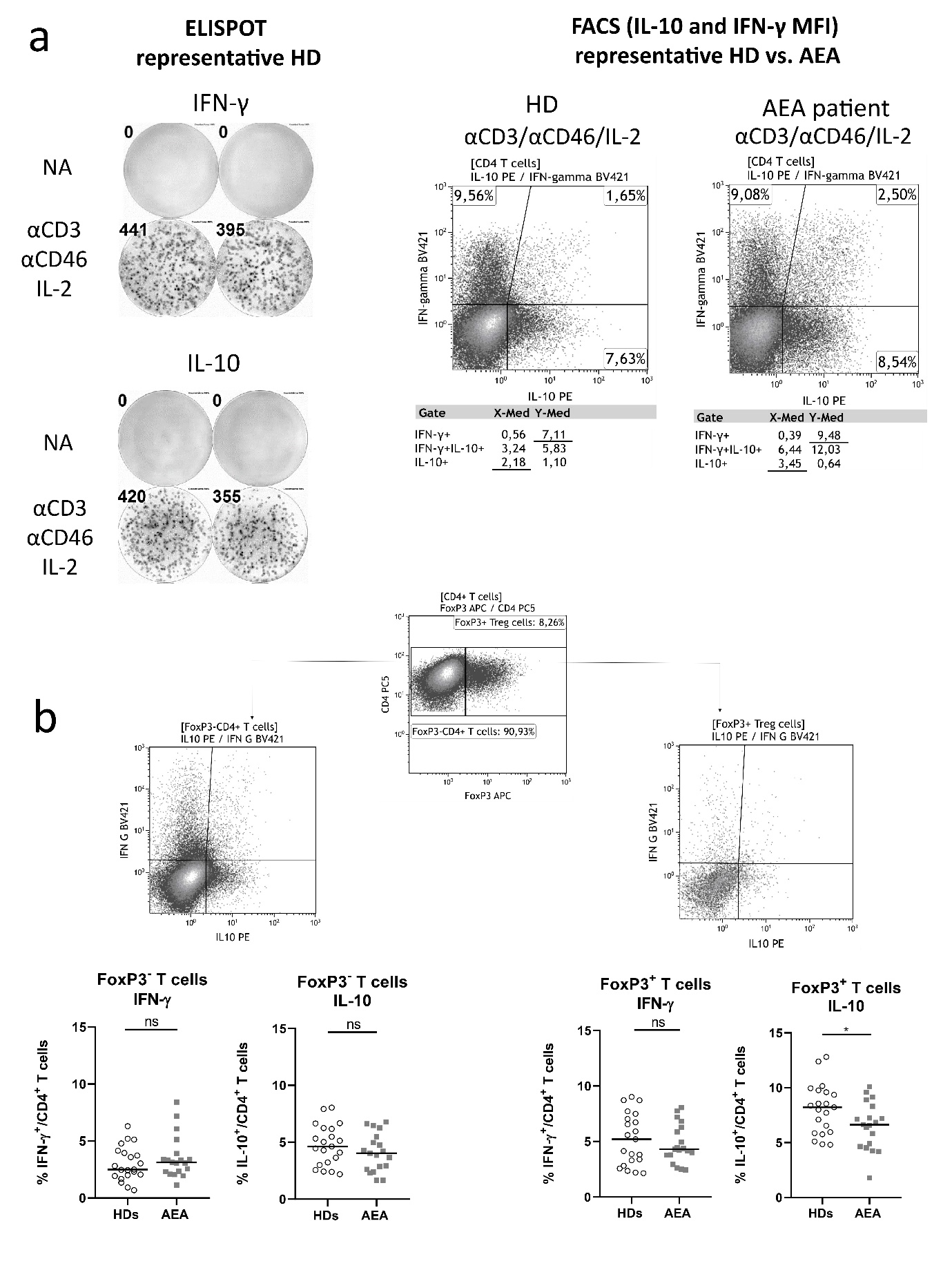
**

(a) A representative example shows an ELISpot IFN-γ and IL-10 production by non-activated (NA) and activated (αCD3/αCD46/IL-2) CD4^+^ T cells from one healthy donor (1/19). FACS dot plots depict differences in IFN-γ and IL-10 MFI despite similar percentages of IFN-γ^+^ and IL-10^+^ CD4^+^ T cells between a representative example of one healthy donor (HD) and patient with AEA. (b) IFN-γ and IL-10 expression was analyzed by flow cytometry separately within CD4^+^FoxP3^+^ and CD4^+^FoxP3^-^ T cells. We observed no difference in proportion of IFN-γ^+^ T cells between HDs and AEA patients, however AEA patients showed decreased frequency of CD4^+^FoxP3^+^IL-10^+^ T cells, indicating an alteration in their functional profile. Dot plots are representative of one healthy donor (1/19). Data were evaluated using the non-parametrical Mann-Whitney U test. The horizontal bar in graphs (b) represents the median. ns (not significant), *p ≤ 0.05. NA, non-activated; HD, healthy donor; AEA, allergic eosinophilic asthma; FACS, fluorescent activated cell sorting; FoxP3, Forkhead box 3.

**Figure S4: Intracellular CD46 expression and soluble CD46 in plasma**

**
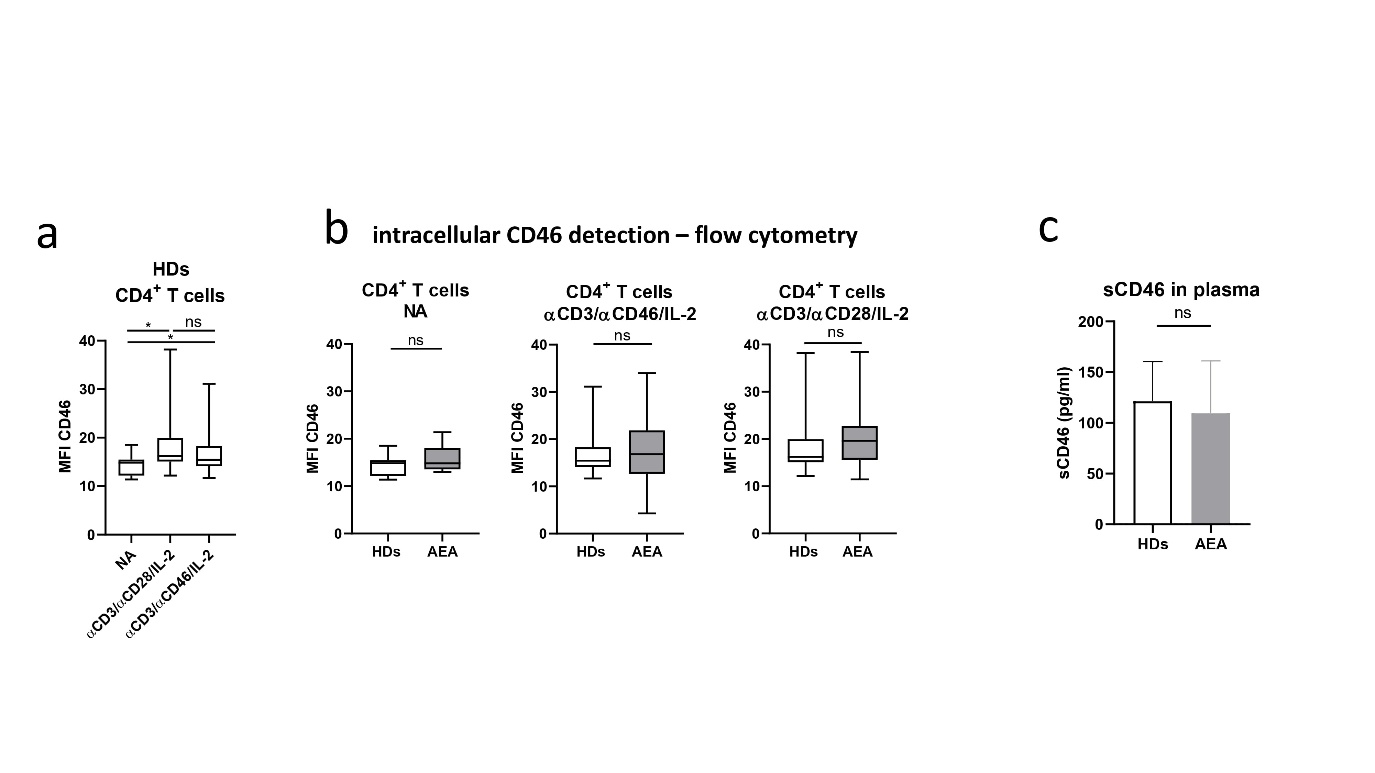
**

(a) Following stimulation (αCD3/αCD28/IL-2 or αCD3/αCD46/IL-2), CD46 was slightly upregulated intracellularly in HDs. Data were evaluated using the Kruskall-Wallis test with Dunn’s correction. (b) However, there was no difference in intracellular CD46 expression between HDs and AEA patients in all conditions and (c) no difference in soluble CD46 (sCD46) concentration in plasma samples. Graphs a and b are depicted as box & whiskers (median, min-max), whereas graph C as median+95% CI. Data were analyzed using the non-parametric Mann-Whitney U test and were evaluated from a smaller group of 19 HDs and 20 AEA patients with moderate AEA. ns (not significant), *p ≤ 0.05. HDs, healthy donors; AEA, allergic eosinophilic asthma; CI, confidence interval.

**Figure S5: Activation marker CD25 and proliferation marker Ki-67 in CD4^+^ T cells activated** **by αCD3/αCD46/IL-2 *in vitro***

**
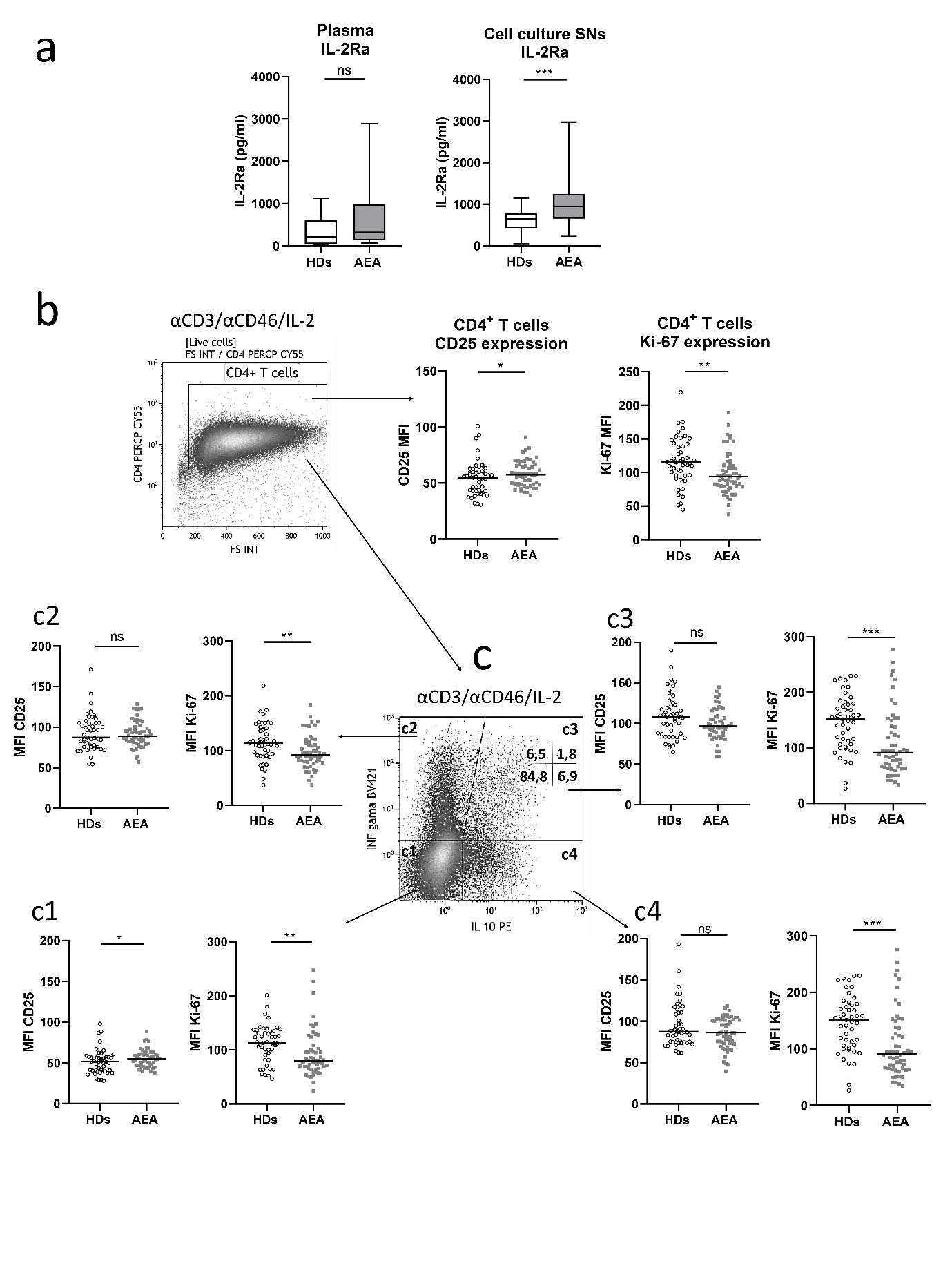
**

(a) IL-2R α chain concentration was evaluated in plasma and cell culture SNs. AEA patients exhibited a borderline increase of IL-2R α chain in plasma and significant elevation in cell culture SNs when compared with HDs following αCD3/αCD46/IL-2 stimulation. Data are presented as box & whiskers (median, min-max). (b) Expression of CD25 and proliferation marker Ki-67 were measured in αCD3/αCD46/IL-2 stimulated CD4^+^ T cells using flow cytometry. Total CD4^+^ T cells showed increased surface expression of CD25 but conversely decreased proliferation in AEA patients. (c) Analysis of CD25 and Ki-67 expression in relation to CD4^+^ T cell function, as indicated by IFN-γ and IL-10 production, revealed that CD25 expression was elevated exclusively in the dominant subset that produced neither cytokine (CD4^+^IFN-γ⁻IL-10⁻) in AEA patients. However, proliferation was reduced across all subsets, with a more pronounced decrease observed in IL-10⁺ subsets (IL-10^-^ subsets p<0.0025 vs. IL-10^+^ subsets p<0.0001) in AEA patients. The horizontal bars in graphs (b) and (c) represent the median. Data were analyzed using the non-parametrical Mann-Whitney U test. ns (not significant), *p ≤ 0.05, **p ≤ 0.01, ***p ≤ 0.001. HDs, healthy donors; AEA, allergic eosinophilic asthma; SNs, supernatants.

**Figure S6: FoxP3^+^ T cell frequency and LAG-3/CD49b expression**

**
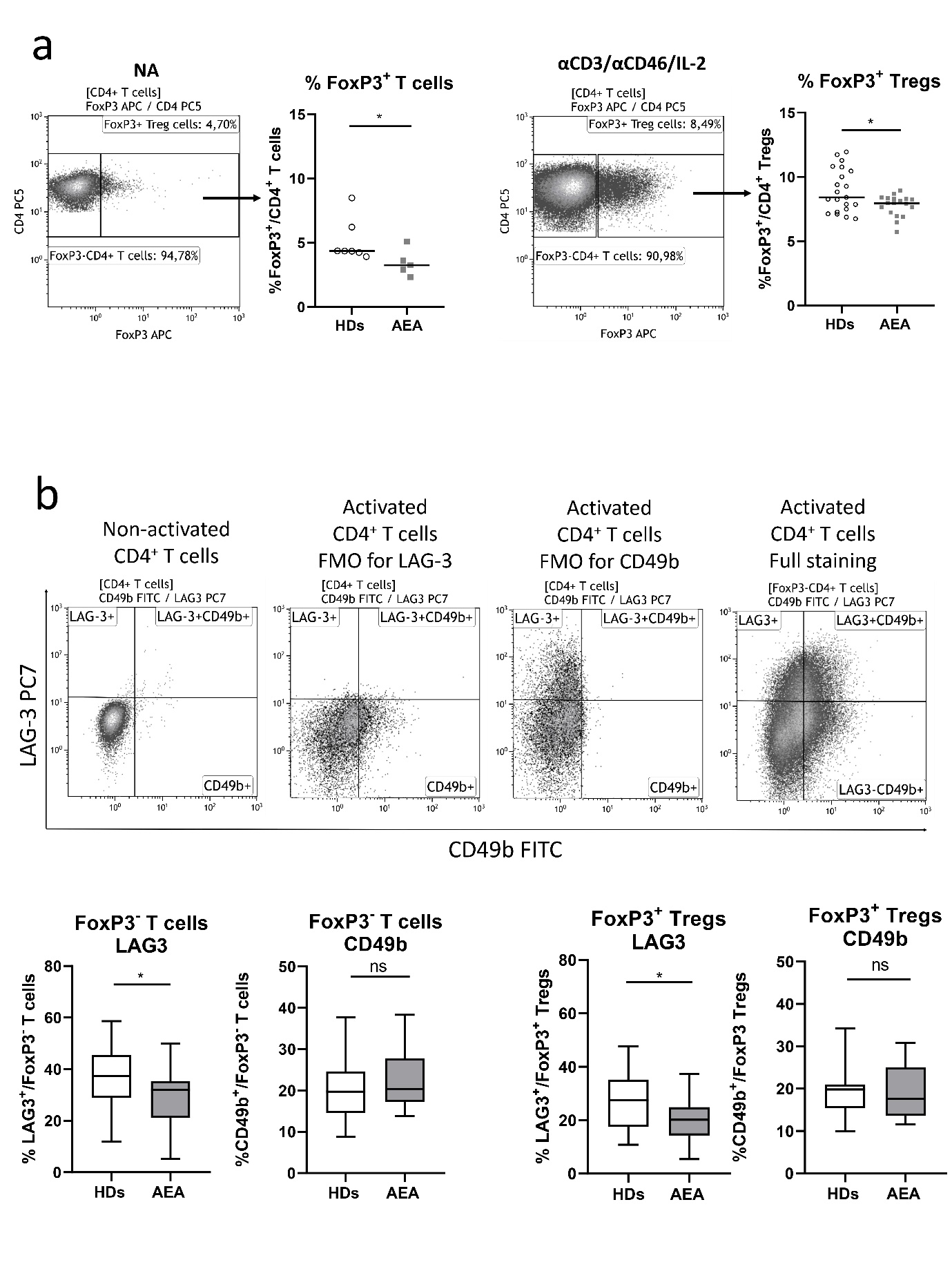
**

(a) Due to limited amount of samples, FoxP3^+^ T cells without stimulation were measured in 7 HDs and 5 AEA patients. FoxP3^+^ T cell proportion after αCD3/αCD46/IL-2 stimulation was assessed in 19 HDs and 20 AEA patients. Both results showed decreased frequency of CD4^+^FoxP3^+^ T cells. (b) Representative dot plots from healthy donor show expression of LAG-3 and CD49b on non-activated CD4+ T cells, fluorescence minus-one (FMO) controls on activated (αCD3/αCD46/IL-2) CD4+ T cells followed by complete staining with both markers. A percentage of LAG-3^+^ cells was decreased in both FoxP3^-^ and FoxP3^+^ T cells from AEA patients. However, CD49b expression was comparable between HDs and AEA patients. Data are presented as box & whiskers (median, min-max) and were evaluated using an unpaired T-test. Results were obtained from a group of 19 HDs and 20 patients with moderate AEA. ns (not significant), *p ≤ 0.05. HDs, healthy donors; AEA, allergic eosinophilic asthma; LAG-3, lymphocyte activation gene 3; FoxP3, forkhead box 3; MFI, median fluorescence intensity; PE, phycoerythrin.

**Supplementary Table S1: Studied subjects characteristics**

Table describes characteristics of healthy donors and patients with allergic eosinophilic asthma, including present comorbidities and applied concomitant therapies. F, female; M, male; x, no corticosteroid therapy.

**Supplementary Table S2: Antibodies used for CD4^+^ T cell staining - flow cytometry**

mAb, monoclonal antibody; FITC, Fluorescein Isothiocyanate; PE, Phycoerythrine; APC, Allophycocyanin; AF-A700, Alexa Fluor 700, APC-A750, Allophycocyanin-alexa 750; ECD, Phycoerythrine-Texas Red; KrO, Krome Orange; PB450, Pacific Blue 450; PC5.5, Phycoerythrine-Cyanine 5.5; PC7, Phycoerythrine-Cyanine 7, APC-Cy7, Allophycocyanine-Cyanine 7; Pe-Dy-Light594, Phycoerythine-DyLight 594; PO, Pacific Orange; BV421, Brilliant Violet 421.
